# MRCK-1 activates non-muscle myosin for outgrowth of a unicellular tube in *Caenorhabditis elegans*

**DOI:** 10.1242/dev.202772

**Published:** 2024-11-29

**Authors:** Evelyn M. Popiel, Rhea Ahluwalia, Stefan Schuetz, Bin Yu, W. Brent Derry

**Affiliations:** ^1^ Developmental and Stem Cell Biology Program, The Hospital for Sick Children, Peter Gilgan Centre for Research and Learning, 686 Bay Street, Toronto, ON M5G 0A4, Canada; ^2^ Department of Molecular Genetics, University of Toronto, 1 King's College Circle, Toronto, ON M5S 1X5, Canada; ^3^ Ontario Institute for Cancer Research, 661 University Avenue, Toronto, ON M5G 0A3, Canada

**Keywords:** MRCK-1, MLC-4, Non-muscle myosin, Outgrowth, Tubulogenesis, *C. elegans*

## Abstract

The formation and patterning of unicellular biological tubes is essential for metazoan development. It is well established that vascular tubes and neurons use similar guidance cues to direct their development, but the downstream mechanisms that promote the outgrowth of biological tubes are not well characterized. We show that the conserved kinase MRCK-1 and its substrate the regulatory light chain of non-muscle myosin, MLC-4, are required for outgrowth of the unicellular excretory canal in *C. elegans*. Ablation of MRCK-1 or MLC-4 in the canal causes severe truncations with unlumenized projections of the basal membrane. Structure-function analysis of MRCK-1 indicates that the kinase domain, but not the small GTPase-binding CRIB domain, is required for canal outgrowth. Expression of a phosphomimetic form of MLC-4 rescues canal truncations in *mrck-1* mutants and shows enrichment at the growing canal tip. Moreover, our work reveals a previously unreported function for non-muscle myosin downstream of MRCK-1 in excretory canal outgrowth that may be conserved in the development of seamless tubes in other organisms.

## INTRODUCTION

Multicellularity necessitates the transport of liquids and gases throughout the organism, and, as such, the formation and maintenance of biological tubes is an essential process for metazoans and many other multicellular organisms. Large tubes comprise multiple cells surrounding an extracellular lumen, while the smallest tubes comprise a single cell where the lumen is contained within the cytoplasm. Depending on their mechanism of formation, unicellular tubes may have an autocellular junction (‘seamed’ tubes) or completely lack tight and adherens junctions (‘seamless’ tubes). Whether they are multicellular or unicellular, biological tubes develop through evolutionarily conserved processes: extracellular guidance cues, definition of apical-basal polarity, vesicle trafficking and cytoskeletal remodeling (Herbert and Stainier, 2011; Lubarsky and Krasnow, 2003; Pradhan et al., 2023; Sundaram and Cohen, 2017; Weinstein, 2005; Xu and Cleaver, 2011). These processes must work in concert to transform single or multiple cells into a functional tube, and their dysregulation can cause abnormal tube formation and a variety of human diseases.

**Fig. 1.**
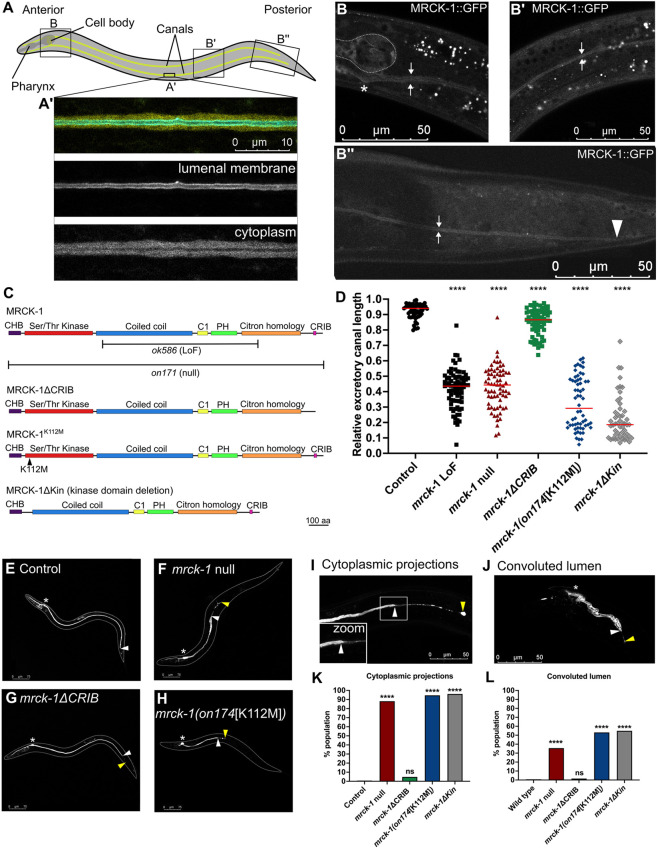
**Loss of *mrck-1* or mutation of its kinase domain causes severe truncations and morphological defects in the excretory canal of adult worms.** (A) The excretory cell of *C. elegans* is a H-shaped unicellular tube that extends canals anteriorly and posteriorly along the length of the body. (A′) Confocal microscopy image of a section of the canal in a wild-type worm with the apical/luminal membrane in cyan (canalp::ifb-1::CFP) and the cytoplasm in yellow (canalp::YFP). (B-B″) A translational reporter encoding MRCK-1::GFP can be seen in the cytoplasm of the excretory canal in the head (B), midbody (B′) and tail (B″)*.* Microscopy images often capture only one canal because the two canals are in different *z*-planes. The bright white puncta are autofluorescence from the lysosome-related gut granules in the intestine and do not represent MRCK-1 expression. The asterisk indicates the cell body of the canal; the dotted line indicates the posterior bulb of the pharynx; white arrows highlight the basal membranes of the canal; the white arrowhead indicates the end of the canal. (C) Structure of wild-type MRCK-1 and the predicted protein products of *mrck-*1*ΔCRIB*, *mrck-1(on174*[K112M]*)* and *mrck-1ΔKin* alleles. *ok586* and *on171* show the *mrck-1(ok586)* loss of function (LoF) and *mrck-1(on171)* null alleles in reference to the wild-type MRCK-1 amino acid sequence. CHB, capped helix bundle; C1, protein kinase C conserved domain 1; PH, pleckstrin homology; CRIB, Cdc42 and Rac-interactive binding. (D-H) *mrck-1* loss-of-function (LoF) (*n*=77), *mrck-1* null (*n*=68), *mrck-1(on174*[K112M]*)* (*n*=52) and *mrck-1ΔKin* (*n*=51) mutants have severe canal truncations, while *mrck-1ΔCRIB* (*n*=71) mutants have modest truncations. Red lines in the graph indicate the medians, ****P<0.0001 versus wild-type control (*n*=58) (Mann–Whitney test). (I-L) A significant proportion of *mrck-1* null, *mrck-1(on174*[K112M]*)* and *mrck-1ΔKin* mutants show a failure of the lumen to extend to the end of the canal (I,K; cytoplasmic projections) and a folding up of the lumen within the cytoplasm of the cell (J,L; convoluted lumen). *n*≥50 for each genotype (see Table S3 for details). ns, *P*≥0.05; ****P<0.0001 versus wild-type control (Fisher's exact test). (E-J) Asterisks indicate the location of the cell body of the excretory cell; white arrowheads indicate the end of the lumenized region of the canal; yellow arrowheads indicate the end of the canal cytoplasm. Controls are wild-type worms carrying the *exc-9::mCherry::rab-11* canal marker (BK205).

Due to their simplicity, seamless tubes provide an ideal model for interrogating fundamental mechanisms of biological tube formation. Seamless tubes are found throughout the animal kingdom, where they make up parts of the vertebrate microvasculature (Bär et al., 1984; Kamei et al., 2006; Wolff and Bär, 1972), as well as parts of organs such as the tip cells of the *Drosophila* trachea (Samakovlis et al., 1996) and the *C. elegans* excretory system (Kenneth Nelson et al., 1983)*.* The largest tube in the excretory system of *C. elegans*, called the excretory canal cell, has been extensively studied to understand the molecular mechanisms of tube formation. The excretory canal is a seamless tube that maintains osmotic balance in the worm (Nelson and Riddle, 1984). This H-shaped cell has its cell body beneath the posterior bulb of the pharynx and extends two projections (canals) anteriorly and posteriorly along the sides of the body and span its entire length (Fig. 1A). Excretory canals are lumenized and connect to the central luminal space in the cell body, which connects to the adjacent duct cell (Kenneth Nelson et al., 1983). The excretory canal cell is born before ventral enclosure during embryogenesis, and by the 1.5-fold stage the lumen of the canal has formed (Stone et al., 2009; Sulston et al., 1983). From this stage of embryogenesis until the end of the first larval molt, the canal undergoes outgrowth as the anterior and posterior branches extend from the cell body and follow guidance cues to reach their target positions at the distal ends of the worm (Fujita et al., 2003). For the remainder of development, the canal is in maintenance phase, growing at the same rate as the rest of the body to maintain its relative position.

During outgrowth and maintenance, the canal requires definition of apical-basal polarity partnered with extensive remodeling of the cytoskeleton and dynamic vesicle trafficking. The apical (luminal) and basal domains of the tube are defined by the PAR polarity proteins for polarized fusion of vesicles to the correct membrane (Abrams and Nance, 2021; Armenti et al., 2014a). Small GTPases, including RAL-1 and CDC-42, are essential regulators of trafficking and polarity in the excretory canal through regulation of the PAR proteins and the exocyst complex (Abrams and Nance, 2021; Armenti et al., 2014a; Lant et al., 2015; Mattingly and Buechner, 2011). Upstream regulators of vesicle trafficking, including CCM3/STRIPAK and the EXC-9/EXC-1/EXC-5(GEF) signaling cascade have also been identified as essential components of canal extension (Armenti et al., 2014a; Gao et al., 2001; Grussendorf et al., 2016; Lant et al., 2015; Suzuki et al., 2001; Tong and Buechner, 2008). Cytoskeletal components, including actin, intermediate filaments, microtubules, and the cytoskeletal linker proteins ERM-1 (ezrin-radixin-moesin) and SMA-1/β-H-spectrin, are required for structural support and to regulate vesicle trafficking at the luminal membrane of the canal (Al-Hashimi et al., 2018; Fujita et al., 2003; Göbel et al., 2004; Khan et al., 2013, 2019; Kolotuev et al., 2013; Shaye and Greenwald, 2015; Woo et al., 2004).

Previously, our lab identified the serine/threonine kinase *mrck-1* (myotonic dystrophy-related Cdc42-binding kinase homolog 1) as a regulator of canal extension downstream of the cerebral cavernous malformation 3 gene *ccm-3*, but the role of MRCK-1 in the canal remains largely uncharacterized (Lant et al., 2015). The myotonic dystrophy-related Cdc42-binding kinases (MRCKs) are highly conserved in animals, including humans, where there are three paralogs: MRCKɑ, MRCKβ and MRCKγ (Unbekandt and Olson, 2014). These kinases are part of the larger dystrophia myotonica protein kinase (DMPK) family, all members of which promote the activation of the actomyosin complex through phosphorylation of the regulatory light chain of non-muscle myosin (Amano et al., 1996; Leung et al., 1998; Luo et al., 1997; Murányi et al., 2001; Yamashiro et al., 2003; Zhao et al., 1997). In vertebrate models, the MRCKs regulate cell-cell junction remodeling, apical polarity and cell migration through actomyosin (Ando et al., 2013; Gomes et al., 2005; Huo et al., 2011; Tan et al., 2011; Zihni et al., 2017); MRCK has been shown to interact with the small GTPase Cdc42 through its Cdc42 and Rac-interactive binding (CRIB) domain (Leung et al., 1998; Luo et al., 1997). MRCKβ has been implicated in lumen formation of endothelial cells in 3D culture downstream of Cdc42, but the mechanism by which it contributes to this process is unknown (Barry et al., 2016; Koh et al., 2008; Norden et al., 2016). In *C. elegans*, *mrck-1* promotes phosphorylation of the regulatory light chain homolog MLC-4 during embryonic elongation (Gally et al., 2009) and functions with active CDC-42 to activate non-muscle myosin during early gastrulation (Marston et al., 2016).

To expand upon our discovery of *mrck-1* as a component of seamless tube formation, we aimed to define the role of MRCK-1 in excretory canal development and to identify the downstream pathway(s) it regulates in this role. Here, we show that the MRCK-1 kinase domain, but not its CRIB domain, is required for canal outgrowth, and that MRCK-1 functions autonomously to promote this process. Mechanistically, we show that phosphorylated MLC-4 acts downstream of *mrck-1* to rescue canal defects in *mrck-1* mutants, and that a phosphomimetic form of MLC-4 is enriched at the tip of the canal during outgrowth. Additionally, we demonstrate a requirement for MLC-4 during excretory canal outgrowth, which phenocopies the canal defects caused by loss of MRCK-1. This work defines a previously unreported role for MRCK-1/non-muscle myosin in seamless tube outgrowth, which may be conserved in other contexts, such as vertebrate tubulogenesis.

## RESULTS

### The MRCK-1 kinase domain is required for excretory canal extension

Using a previously published translational reporter of MRCK-1, we were able to confirm its expression in the excretory canal of adult worms (Fig. 1B-B″). We observed expression of MRCK-1::GFP in the cytoplasm of the canal throughout its entire length (Fig. 1B-B″). MRCK-1::GFP expression was seen in additional tissues, including the pharynx (Fig. 1B).

Given the established role of the MRCKs as kinases and Cdc42-binding proteins (Leung et al., 1998; Luo et al., 1997), we tested the requirement of the kinase and CRIB domains for MRCK-1 function in the excretory canal. Using CRISPR/Cas9 gene editing, we edited *mrck-1* at its endogenous locus to create a CRIB domain deletion allele (*mrck-1ΔCRIB*) that removes the last 58 amino acids of the protein containing the 14 amino acid CRIB domain (Fig. 1C). We created a missense allele that encodes a K112M amino acid substitution in the kinase domain of MRCK-1 (*mrck-1(on174*[K112M]*)*), which is predicted to abrogate kinase activity by disrupting ATP-binding (Fig. 1C) (Sumi et al., 2001; Wilkinson et al., 2005). Additionally, we created a kinase domain deletion allele (*mrck-1ΔKin*) that encodes an in frame 1350 bp deletion that removes the entire kinase domain of MRCK-1 (Fig. 1C). We also created a *mrck-1* null allele by deleting the entire coding sequence of the gene (Fig. 1C), as a positive control for complete loss of MRCK-1. Additionally we analyzed a published *mrck-1* loss-of-function allele (*ok586*) that we used previously to test the requirement of *mrck-1* for canal extension (Lant et al., 2015; The C. elegans Deletion Mutant Consortium, 2012). The *mrck-1* loss-of-function allele encodes a complex substitution (2447 bp deletion and 124 bp insertion) predicted to cause splice site variants and missense mutations in the protein that abolish its function.

While excretory canals of adult wild-type worms extend almost to the end of the worm (median=0.94 relative length), we found that the *mrck-1* null, *mrck-1[K112M]* and *mrck-1ΔKin* mutants had severely truncated canals that were less than half the body length of the worm (median=0.44, 0.26, and 0.19, respectively) (Fig. 1D,F,H, Fig. S1B). The *mrck-1ΔCRIB* mutants also had canals that were significantly shorter than wild type, but the degree of truncation was mild compared with the other *mrck-1* mutants (median 0.87) (Fig. 1D,G). We found that there is no significant difference between the canal lengths of *mrck-1* loss-of-function and *mrck-1* null mutants, so we conclude that the loss-of-function allele causes complete loss of *mrck-1* function (Fig. 1D).

The canals of the *mrck-1* null, *mrck-1[K112M]* and *mrck-1ΔKin* mutants show two other morphological defects that were absent in wild-type and *mrck-1ΔCRIB* mutant worms: cytoplasmic projections and convoluted lumens. Cytoplasmic projection is a defect in which the basal membrane and cytoplasm of the canal extend without the luminal membrane, creating an unlumenized section at the tip of the canal (Fig. 1I) (Khan et al., 2013; Lant et al., 2015). Cytoplasmic projections were observed in fewer than 1% of wild-type worms and only 6.7% of *mrck-1ΔCRIB* mutants, but occurred in the majority of *mrck-1* null, *mrck-1[K112M]* and *mrck-1ΔKin* mutant populations (88.7%, 95.0% and 96%, respectively) (Fig. 1K). Convoluted lumen is a defect in which the lumen of the canal appears ‘folded up’ in the cytoplasm of the cell (Fig. 1J) (Buechner, 2002; Buechner et al., 1999), which was observed in fewer than 2% of wild-type worms and *mrck-1ΔCRIB* mutants, but was frequent in *mrck-1* null, *mrck-1[K112M]* and *mrck-1ΔKin* mutants (37.1%, 53.4% and 54.9%) (Fig. 1K).

Overall, the similarities in canal truncations and other morphological defects between the *mrck-1* null, *mrck-1[K112M]* and *mrck-1ΔKin* mutants suggests that the kinase domain is essential for MRCK-1 function in the canal. The mild canal truncations and rare morphological defects in the *mrck-1ΔCRIB* mutants suggests that this domain, and potentially an interaction with CDC-42, is not necessary for MRCK-1 function in canal extension.

### Maternally supplied *mrck-1* promotes canal extension

**Fig. 2.**
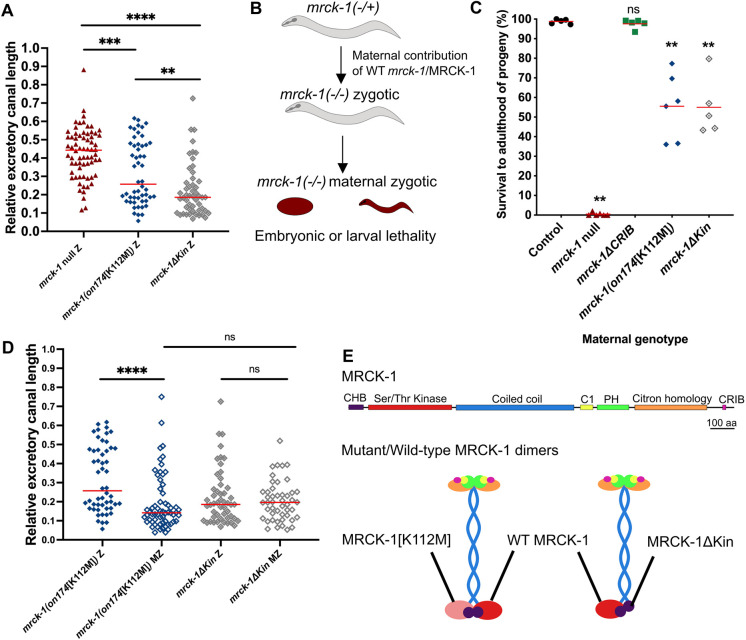
**Maternally supplied *mrck-1*/MRCK-1 suppresses canal truncations in *mrck-1* null and *mrck-1[K112M]* mutants.** (A) *mrck-1(on174*[K112M]*)* zygotic (Z) mutants (*n*=52) have significantly shorter canals than *mrck-1* null Z mutants (*n*=68), while *mrck-1ΔKin* Z mutants (*n*=51) have significantly shorter canals than *mrck-1* null or *mrck-1(on174*[K112M]*)* Z mutants. **P<0.01, ***P<0.001, ****P<0.0001 (Mann–Whitney test). Red lines indicate medians. (B) *mrck-1* Z mutants survive embryonic elongation due to maternally contributed wild-type *mrck-1*/MRCK-1, while *mrck-1* maternal zygotic (MZ) mutants die as embryos or L1s. (C) While the survival of *mrck-1* null MZ mutants is less than 1%, a mean of 57% of *mrck-1(on174*[K112M]*)* and 55.0% *mrck-1ΔKin* MZ mutants survive to adulthood. ns, *P*≥0.05; **P<0.01 versus wild-type control (Welch's *t*-test). Red lines indicate means. (D) The canals of *mrck-1(on174*[K112M]*)* MZ mutants (*n*=52) are significantly shorter than the Z mutants (*n*=62), while there is no significant difference between the canal lengths of *mrck-1ΔKin* Z or MZ mutants. ****P<0.0001 (Mann–Whitney test). Red lines indicate medians. (E) Schematic of MRCK-1 protein domains and the 3D structure of MRCK-1 dimers, with the corresponding domains in matching colors. CHB, capped helix bundle; C1, protein kinase C conserved domain 1; PH, pleckstrin homology; CRIB, Cdc42 and Rac-interactive binding. Mutant proteins encoded by the *mrck-1(on174*[K112M]*)* and *mrck-1ΔKin* alleles may form dimers with maternally contributed wild-type MRCK-1. Controls are wild-type worms carrying the *exc-9::mCherry::rab-11* canal marker (BK205).

When quantifying the canal truncations of the various *mrck-1* mutants, we observed that the *mrck-1[K112M]* and *mrck-1ΔKin* mutants had significantly shorter canals than the *mrck-1* loss-of-function or null mutants (Fig. 2A). Additionally, we observed that the canals of the *mrck-1ΔKin* mutants were significantly shorter than the *mrck-1[K112M]* mutants (Fig. 2A). We hypothesized that the less severe truncations observed in *mrck-1* null mutants may be due to maternal rescue. In addition to its function in the canal, *mrck-1* is required during embryogenesis for the activation of non-muscle myosin during embryonic elongation (Gally et al., 2009). Zygotic (Z) *mrck-1* mutants from heterozygous mothers survive to adulthood due to maternally contributed wild-type *mrck-1*, while the maternal zygotic (MZ) *mrck-1* mutants die during embryogenesis or at the first larval stage (Fig. 2B). We measured the maternal embryonic/larval lethal phenotype of the *mrck-1* alleles we generated as the survival to adulthood of MZ progeny (percent of total progeny laid) and compared this to wild-type controls (Fig. 2C). Almost all progeny from wild-type control mothers survive to adulthood, compared to less than 1% of progeny from *mrck-1* null mutants (Fig. 2C). The *mrck-1[K112M]* and *mrck-1ΔKin* mutants had an intermediate phenotype, with about half of the progeny surviving to adulthood (mean survival 57% and 55%, respectively) (Fig. 2C). All the previously analyzed *mrck-1* mutants were Z mutants, apart from the *mrck-1ΔCRIB* mutants, which were maintained as homozygotes due to their wild-type level of survival to adulthood (Fig. 2C). Since the *mrck-1[K112M]* and *mrck-1ΔKin* MZ mutants can survive to adulthood, we compared the relative canal lengths of these mutants to the previously characterized Z mutants (Fig. 2D). We found that the *mrck-1[K112M]* MZ mutants have significantly shorter canals than the Z mutants, while there is no significant difference in the canal lengths of the *mrck-1ΔKin* MZ and Z mutants (Fig. 2D). There is also no significant difference between the canal lengths of the *mrck-1[K112M]* MZ and *mrck-1ΔKin* MZ mutants, which suggests that the MRCK-1[K112M] mutation causes a similar loss of kinase activity to complete deletion of the kinase domain (Fig. 2D). These findings also suggest that maternally supplied wild-type MRCK-1 partially suppresses canal truncations in *mrck-1[K112M]* Z mutants, but not in the *mrck-1ΔKin* Z mutants. Both the alleles are predicted to encode proteins that contain the capped helix bundle (CHB) domain required for MRCK-1 dimerization, so it is possible that the MRCK-1ΔKin protein creates non-functional dimers with the maternally contributed wild-type MRCK-1, while the MRCK-1[K112M]/WT MRCK-1 dimers retain some function (Fig. 2E). The *mrck-1[K112M]* and *mrck-1ΔKin* alleles do not function in a dominant-negative manner, as heterozygous mutants have canals that are of wild-type length (Fig. S2). Overall, these findings suggest that maternally contributed *mrck-1* plays a role in excretory canal development and indicate that the effect of total loss of *mrck-1* is partially suppressed in the previously quantified *mrck-1* null Z mutants.

### *mrck-1* is required for canal outgrowth

**Fig. 3.**
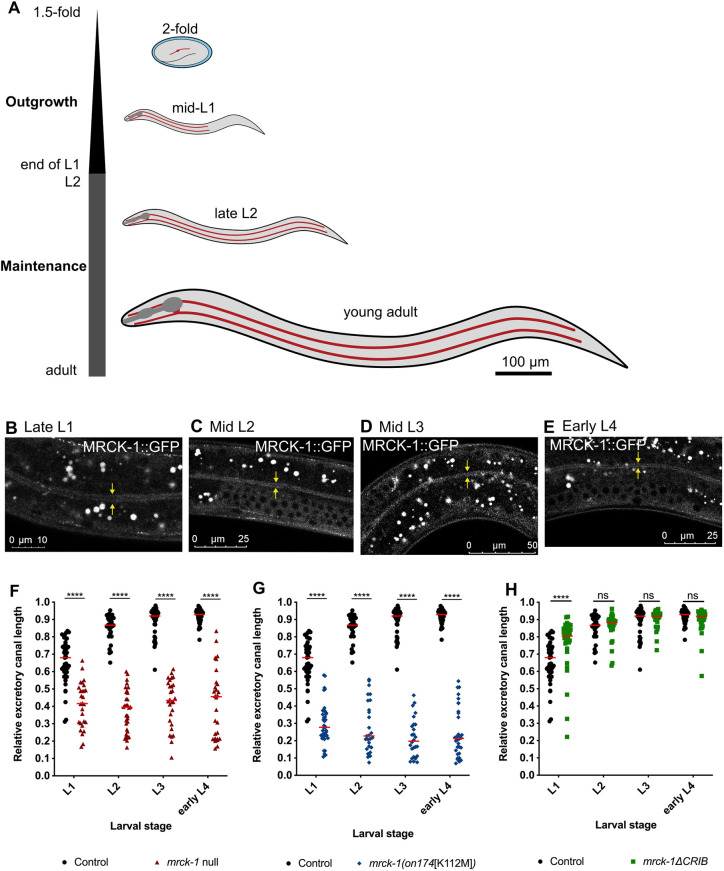
***mrck-1* and its kinase domain are required for excretory canal outgrowth.** (A) From the 1.5-fold stage during embryogenesis to the end of the first larval stage (L1), the excretory canal undergoes outgrowth, where it follows guidance cues to reach its final position near the tail of the worm. For the rest of development, the canal undergoes maintenance, where it grows at the same rate as the body to maintain its relative position. Embryo is not to scale. (B-E) The MRCK-1 translational reporter can be seen in the canal at each larval stage. The yellow arrows highlight the basal membranes of the canal. The bright white puncta are autofluorescence from the lysosome-related gut granules in the intestine and do not represent MRCK-1 expression*.* (F,G) The *mrck-1* null (F) and *mrck-1(on174*[K112M]*)* (G) mutants have severe canal truncations by L1, and these defects persist throughout development. (H) *mrck-1ΔCRIB* mutants do not have canals shorter than wild-type controls at any stage of larval development. Sample sizes (L1, L2, L3 and eL4): *mrck-1* null, *n*=28, 32, 28 and 28; *mrck-1(on174*[K112M]*)*, *n*=39, 27, 26 and 32; *mrck-1ΔCRIB*, *n*=34, 29, 27 and 35; control, *n*=44, 30, 39 and 33. Red lines in the graphs indicate the medians. ns, *P*≥0.05; ****P<0.0001 versus wild-type control (Mann–Whitney test). Controls are wild-type worms carrying the *exc-9::mCherry::rab-11* canal marker (BK205).

The previous evaluation of canal lengths in *mrck-1* mutants was performed on late L4 stage and early adult worms when the canal has fully developed. To gain insight into the timing of *mrck-1* function during development, we next investigated its expression and function during larval stages. The development of the excretory canal can be divided into two discrete phases: outgrowth and maintenance (Fig. 3A). Outgrowth begins immediately after the lumen is formed at the 1.5-fold stage of embryogenesis and lasts until the end of the first larval stage, when the anterior and posterior canal tips follow molecular guidance cues that extend the canals to reach their target positions (Fig. 3A). After this, the canals switch to a maintenance phase where they grow at the same rate as the body to maintain their relative position (Fig. 3A) (Buechner, 2002; Fujita et al., 2003).

Using the same translational reporter as before, we were able to confirm expression of MRCK-1 in the canal from late L1 stage to early L4 stage (Fig. 3B-E). MRCK-1::GFP can be seen throughout the cytoplasm in the anterior, midbody and posterior sections of the canal during each larval stage (Fig. S3).

Next, we compared the relative canal lengths of *mrck-1* null, *mrck-1[K112M]* and *mrck-1ΔCRIB* mutants to wild-type worms at each larval stage to determine when truncations first appear (Fig. 3F-H). Due to the phenotypic similarities in canal defects and MZ canal truncations of the *mrck-1[K112M]* and *mrck-1ΔKin* mutants, we only analyzed the canals of *mrck-1[K112M]* mutants during larval development. The *mrck-1* null and *mrck-1[K112M]* mutants have significantly shorter canals than wild-type controls by the first larval stage, and these truncations persist throughout development (Fig. 3F,G). This suggests that *mrck-1* kinase activity is required during the outgrowth phase of canal development. *mrck-1ΔCRIB* mutants do not have shorter canals than wild-type worms at any larval stage, which suggests that an interaction with CDC-42 is dispensable for MRCK-1 function during canal outgrowth and much of the maintenance phase (excluding late L4/early adult) (Fig. 3H).

### MRCK-1 functions autonomously in the canal for outgrowth

**Fig. 4.**
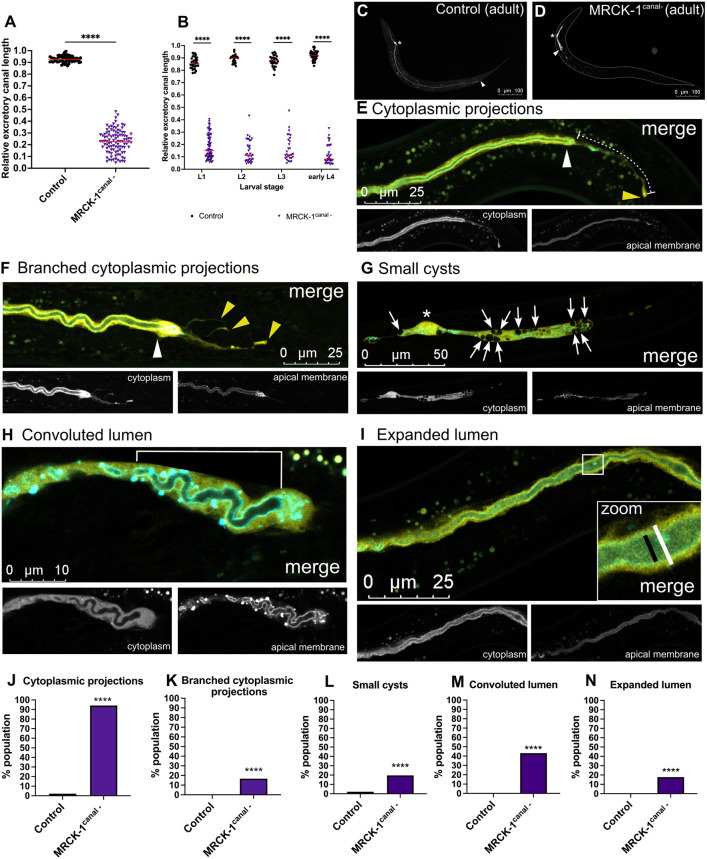
**MRCK-1 functions autonomously in the excretory canal cell during outgrowth.** (A,C,D) MRCK-1^canal-^ adults (*n*=102) with MRCK-1 ablated only in the excretory canal cell have extremely truncated canals that are significantly shorter than wild-type controls (*n*=95). ****P<0.0001 (Mann–Whitney test). Asterisks indicate the location of the cell body of the excretory cell; white arrowheads indicate the end of the lumenized region of the canal. (B) By the first larval stage, the canals of MRCK-1^canal-^ mutants are significantly shorter than wild-type controls, and these truncations persist throughout development. Sample sizes (L1, L2, L3 and eL4): MRCK-1^canal-^, *n*=66, 33, 25 and 19; control, *n*=34, 28, 30 and 44. ****P<0.0001 (Mann–Whitney test). Red lines on graphs indicate population medians. (E-I) Adult MRCK-1^canal-^ mutants have several morphological defects, including (E) cytoplasmic projections (white dashed line), (F) branched cytoplasmic projections, (G) convoluted lumens (white solid line), (H) small cysts (white arrows) and (I) expanded lumens (black line is the diameter of the lumen; white line is the diameter of the canal). White arrowheads indicate the end of the lumen of the canal; yellow arrowheads indicate the end of the basal membrane. Asterisk indicates the location of the cell body of the excretory cell. (J-N) All these defects were observed at a significantly higher frequency in MRCK-1^canal-^ mutants compared to wild-type controls. ****P<0.0001 (Fisher's exact test), *n*≥95 (see Table S3 for details). Controls are worms carrying the *t28h11.8p::yfp::sl2::ifb-1::cfp* canal marker and *t28h11.8p::zif-1* canal-specific ZIF-1 transgenes (WD982).

To circumvent the maternal contribution of *mrck-1*, we used the tissue-specific ZIF-1/ZF1 protein degradation system, which was previously adapted to degrade target proteins rapidly and specifically in the canal (Abrams and Nance, 2021). This system uses tissue-specific expression of the *C. elegans* substrate-recognition protein ZIF-1 to recruit target proteins tagged with a ZF1 recognition motif to the E3/E2 ubiquitin ligase complexes for poly-ubiquitylation and subsequent proteasome-mediated degradation (Armenti et al., 2014b; 2014a; Sallee et al., 2018). To ablate MRCK-1 specifically in the canal (MRCK-1^canal-^), we added the ZF1 recognition motif sequence to the C-terminus of *mrck-1* at its endogenous locus in a strain with a transgene that expresses ZIF-1 under a canal-specific promoter (Fig. S4A). The adult MRCK-1^canal-^ mutants had canals that were significantly shorter than wild type, demonstrating an autonomous function for MRCK-1 in the canal (Fig. 4A,C,D). MRCK-1^canal-^ mutants also have significantly shorter canals than the *mrck-1* null mutants, providing further evidence that maternally contributed *mrck-1* functions in canal extension (Fig. S5). By measuring canals during larval development, we found that the MRCK-1^canal-^ mutants have canals that are significantly shorter than wild type, beginning at the first larval stage, providing more evidence that MRCK-1 is required for canal outgrowth (Fig. 4B).

The MRCK-1^canal-^ mutants express fluorescent reporters that mark the luminal membrane of the canal with CFP and the cytoplasm with YFP, which enabled identification of morphological defects that were not apparent with the previously used cytoplasm-only canal marker. In addition to cytoplasmic projections and convoluted lumens, we identified three more morphological defects in adult MRCK-1^canal-^ mutants: branched cytoplasmic projections, small cysts and expanded lumens (Fig. 4E-I). Branched cytoplasmic projections are a subtype of cytoplasmic projections defect where the projection at the tip of the canal becomes two or more separate branches (Fig. 4F). Small cysts are spherical structures smaller than the diameter of the canal contained in the cytoplasm of the cell (Fig. 4G). The expanded lumen phenotype describes canals in which the diameter of the canal lumen is greater than 50% of the total canal diameter (Fig. 4I, Fig. S6A). All these defects were observed at a significantly higher frequency in MRCK-1^canal-^ mutants than wild-type controls, where they were seldom or never observed (Fig. 4J-N).

We also quantified the presence of these morphological defects in the canals of the MRCK-1^canal-^ mutants at each larval stage. There was a significant increase in the frequency of expanded and convoluted lumens in the MRCK-1^canal-^ mutants throughout development (Fig. S7D,E), while there was no significant difference in the frequency of cytoplasmic projections (single or branched) or of small cysts (Fig. S7A-C). This suggests that the cytoplasmic projections and cyst defects are a consequence of loss of MRCK-1 during canal outgrowth. The increase in expanded and convoluted lumen defects during development may indicate a requirement for MRCK-1 during the maintenance phase or continued growth of the lumen in truncated canals.

### Expression of a phosphomimetic MLC-4 mutant rescues canal truncations in *mrck-1* mutants

**Fig. 5.**
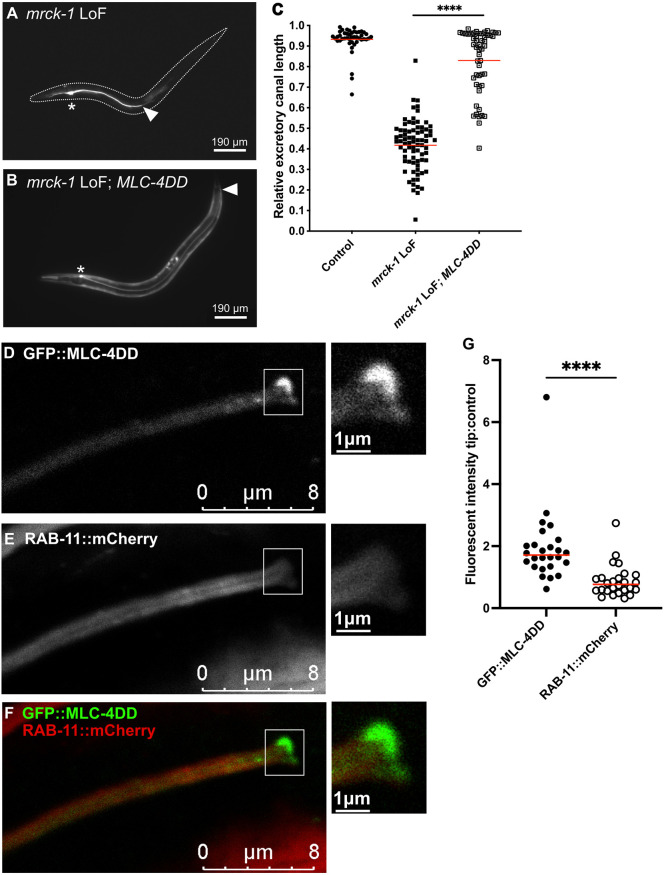
**Expression of a MLC-4 phosphomimetic mutant rescues canal truncations in *mrck-1* mutants.** (A,B) Canal truncations observed in the *mrck-1* loss-of-function mutants (A) can be rescued by overexpression of *mlc-4DD* under the *exc-9* (strong canal promoter) and *mlc-4* promoters as a multicopy extrachromosomal array (B). Asterisks indicate the location of the cell body of the excretory cell; white arrowheads indicate the end of the lumenized region of the canal. (C) *mrck-1* mutants with the *mlc-4DD* transgene (*n*=52) have significantly longer canals than the single mutants (*n*=77). Wild-type control *n*=46. ****P<0.0001 (Mann–Whitney test). (D-F) GFP::MLC-4DD expressed in the canal shows enrichment at the growing tip of the canal during outgrowth in an L1 larva. This enrichment at the growing tip was not observed in the control RAB-11::mCherry marker, which has a diffuse cytoplasmic localization. (G) L1 worms expressing *gfp::mlc-4DD* in the canal show enrichment of GFP::MLC-4DD at the tip of the canal, compared to an adjacent control region. RAB-11::mCherry fluorescence is slightly diminished at the canal tip compared to the control region. *n*=26. ****P<0.0001 (paired samples Wilcoxon test). Red lines on graphs indicate medians. Controls are *mrck-1* loss-of-function heterozygotes carrying the *mlc-4DD* transgene (WD509) (*mrck-1* loss-of-function heterozygotes have wild-type canals).

Since the requirement of the kinase domain supports a function for MRCK-1 kinase activity in the canal, we next wanted to identify genes that could be downstream kinase substrates. We hypothesized that MRCK-1 may function through its known target, the regulatory light chain (RLC) of non-muscle myosin (MLC-4). MRCK-1 is known to promote phosphorylation of MLC-4 during early gastrulation and embryonic elongation to generate contractile force (Gally et al., 2009; Marston et al., 2016). Non-muscle myosin is activated by phosphorylation of two conserved residues on the RLCs, threonine 17 and serine 18 in MLC-4 (Gally et al., 2009). To test for a role for *mlc-4* downstream of *mrck-1* in canal extension, we replaced threonine 17 and serine 18 with aspartic acids (MLC-4DD) to generate a phosphomimetic protein, which was expressed in *mrck-1* loss-of-function mutants under the strong canal promoter *exc-9* as a multicopy extrachromosomal array. Overexpression of the MLC-4DD phosphomimetic mutant was able to rescue canal truncations in the *mrck-1* mutants, which suggests that phosphorylation of MLC-4 functions downstream of MRCK-1 for canal extension (Fig. 5A-C). MRCKs have been shown to phosphorylate the RLC of non-muscle myosin directly (Leung et al., 1998), or through inhibitory phosphorylation of myosin light chain phosphatase (MLCP), which is encoded by *mel-11* in *C. elegans* (Gally et al., 2009; Tan et al., 2001; Wilkinson et al., 2005). To determine whether *mel-11* acts downstream of *mrck-1* in the canal, we knocked down its expression in *mrck-1* null mutants. Knockdown of *mel-11* did not rescue canal truncations in the *mrck-1* mutants, which suggests that *mrck-1* does not inhibit this phosphatase to promote phosphorylation of MLC-4 for canal extension (Fig. S8).

To better understand the function of phosphorylated MLC-4 in the excretory canal, we observed the localization of the MLC-4 phosphomimetic mutant tagged with GFP (GFP::MLC-4DD) expressed under a canal-specific promoter. Localization of this translational reporter was compared with a RAB-11::mCherry reporter expressed in the canal, which shows diffuse localization throughout the cytoplasm. We found that, in L1s, when the canal is in outgrowth stage, GFP::MLC-4DD is enriched at the growing tip of the canal (Fig. 5D,F,G). This enrichment at the growing tip is unique to GFP::MLC-4DD, whereas the RAB-11::mCherry marker is not enriched at the canal tip (Fig. 5E-G).

Activated non-muscle myosin binds to F-actin through the head domain of the heavy chain to form actomyosin, the protein complex that exerts contractile force upon structures within the cell. Given the interaction between actin and non-muscle myosin, and the enrichment of F-actin (Shaye and Greenwald, 2015) and GFP::MLC-4DD at the tip of the canal, we wanted to see whether F-actin organization was perturbed upon loss of *mrck-1*. Localization of F-actin was observed using a LifeAct::TagRFP reporter expressed in the excretory canal. At L1 stage, during canal outgrowth, the LifeAct::TagRFP reporter also showed enrichment at the growing tip in wild-type worms, demarking the previously described leading edge F-actin structure (Shaye and Greenwald, 2015) (Fig. S9A). This localization of F-actin at the leading edge did not change in the *mrck-1* mutants (Fig. S9B), indicating that F-actin organization is not detectably affected by this kinase in the canal.

### MLC-4 functions autonomously to promote canal outgrowth

Since our genetic evidence suggests that *mlc-4* functions downstream of *mrck-1* in the canal, we wanted to determine whether *mlc-4* functioned autonomously to promote canal outgrowth. *mlc-4* is an essential gene due to its roles in cytokinesis and polarity during early embryogenesis (Shelton et al., 1999) and elongation at later stages of embryonic development (Gally et al., 2009). Therefore, to avoid lethality from complete loss of *mlc-4*, we turned to the ZIF-1/ZF1 protein degradation system again to specifically ablate MLC-4 in the canal. We added a start codon and the ZF1 recognition motif sequence upstream of *mlc-4* at its endogenous locus in a strain with a transgene that expresses ZIF-1 under a canal-specific promoter and a loss-of-function mutation in *zif-1* to prevent degradation of MLC-4 in the early embryo (Fig. S4B) (Sallee et al., 2018).

**Fig. 6.**
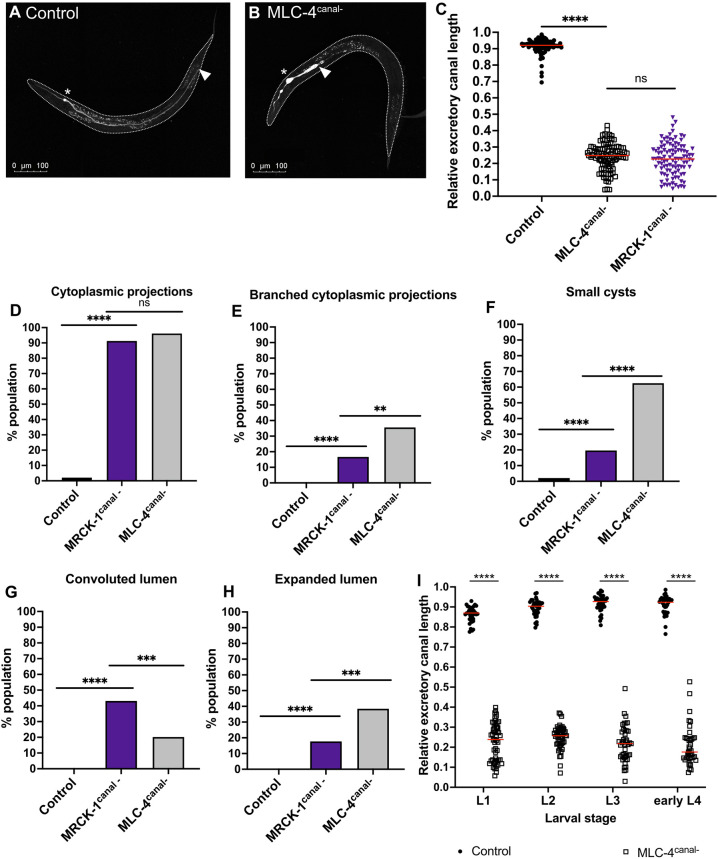
**Ablation of MLC-4 in the excretory cell causes canal truncations and morphological defects that resemble MRCK-1 ablation.** (A-C) MLC-4^canal-^ mutants (*n*=104) have significantly shorter canals than wild-type controls (*n*=91), but there is no significant difference between these mutants and the canals of MRCK^canal-^ mutants (*n*=102). ns, *P*≥0.05; ****P<0.0001 (Mann–Whitney test). Asterisks indicate the location of the cell body of the excretory cell; white arrowheads indicate the end of the lumenized region of the canal. Red lines on graphs indicate population medians. (D-H) MLC-4^canal-^ mutants display the same types of canal defects as MRCK-1^canal-^ mutants. (D) Cytoplasmic projections are present at equal frequencies in MLC-4^canal-^ and MRCK^canal-^ mutants, but the other defects are more frequent in one mutant. (E-G) Branched cytoplasmic projections (E), small cysts (F) and expanded lumens (H) are more prevalent in MLC-4^canal-^ mutants (E,F,H), while convoluted lumens are more prevalent in MRCK^canal-^ mutants (G). MLC-4^canal-^ (*n*=104); MRCK^canal-^ (*n*=102); wild-type controls (*n*=91). ns, *P*≥0.05; **P<0.01, ***P<0.001, ****P<0.0001 (Fisher's exact test). (I) By L1 stage, the canals of MLC-4^canal-^ mutants are significantly shorter than wild-type controls, and these truncations persist throughout development. Sample sizes (L1, L2, L3 and eL4): MLC-4^canal-^ *n*=53, 51, 43 and 43; wild-type control *n*=38, 36, 38 and 36. ****P<0.0001 (Mann–Whitney test). Red lines on graphs indicate population medians. Controls are worms carrying the *t28h11.8p::yfp::sl2::ifb-1::cfp* canal marker, *t28h11.8p::zif-1* canal-specific ZIF-1 transgene and *zif-1(gk117)* loss-of-function mutation (WD1032).

We found that MLC-4^canal-^ mutants had severe canal truncations, supporting an autonomous role for MLC-4 in promoting canal extension (Fig. 6A,B). Notably, the canal lengths of MLC-4^canal-^ and MRCK-1^canal-^ mutants are not significantly different, which is consistent with MLC-4 function downstream of MRCK-1 for canal extension (Fig. 6C). Like MRCK-1^canal-^ mutants, the canals of MLC-4^canal-^ mutants have single and branched cytoplasmic projections, small cysts, convoluted lumens and expanded lumens (Fig. 6D-H). Although MLC-4^canal-^ and MRCK-1^canal-^ mutants have the same types of morphological defects, some of these defects were present at different frequencies. There was no significant difference in the frequency of cytoplasmic projections, as they were present in almost all MLC-4^canal-^ and MRCK-1^canal-^ mutants (Fig. 6D). The branched cytoplasmic projections and expanded lumens were approximately twice as prevalent in MLC-4^canal-^ mutants compared to MRCK-1^canal-^ mutants, while small cysts were three times as prevalent (Fig. 6E,F,H). Conversely, convoluted lumens were twice as prevalent in MRCK-1^canal-^ mutants (Fig. 6G).

Canal truncations in MLC-4^canal-^ mutants were observed in the first larval stage during canal outgrowth and persisted into adulthood (Fig. 6I), which suggests a role for non-muscle myosin in canal outgrowth. Similar to MRCK-1^canal-^ mutants, cytoplasmic projections were observed at a high frequency in MLC-4^canal-^ mutants, starting at L1 stage, and did not significantly change during development (Fig. S10A), which suggests that this defect arises during outgrowth. The frequency of expanded and convoluted lumens was very low to absent in the MLC-4^canal-^ mutants at the L1 stage, and significantly increased throughout development to adulthood (Fig. S10D,E). This pattern is similar to the development of expanded and convoluted lumens in the MRCK-1^canal-^ mutants, and could indicate a requirement for MLC-4 later in development, or a general consequence of extremely truncated canals. Unlike the MRCK-1^canal-^ mutants, the frequency of small cysts significantly increased in MLC-4^canal-^ mutants after the 2nd larval stage (Fig. S10C), which could indicate a role for non-muscle myosin in vesicle trafficking during maintenance phase, in addition to a role during outgrowth.

## DISCUSSION

The formation and patterning of tubes is an essential process for many organs and structures in metazoans, and the molecular mechanisms that govern these processes are highly conserved. Here, we have defined an autonomous role for the conserved kinase MRCK-1 in the development of the seamless excretory canal cell in *C. elegans.* Mutations that disrupt the kinase domain, but not the Cdc42-binding CRIB domain, prevent outgrowth of the canal. Consistent with these observations, previous work has shown that Cdc42 is not required for MRCKA kinase activity (Leung et al., 1998), although most previously characterized functions of MRCK kinases *in vivo* or *in vitro* require Cdc42-dependent localization at specific cell membrane sites for its proper function (Ando et al., 2013; Huo et al., 2011; Marston et al., 2016; Zihni et al., 2017). In the *Drosophila* visual system, the MRCK homolog *gek* is required for axon targeting in a CRIB-independent manner, although the phenotypic similarities between *gek* and *cdc42* mutants led the authors to conclude that this is still a Cdc42-dependent process (Gontang et al., 2011)*.* Ablation of CDC-42 in the excretory canal has been reported to cause less severe truncations that occur later in development than those observed in our *mrck-1*/MRCK-1^canal-^ mutants; these defects are caused by a loss of polarized localization of the exocyst complex (Abrams and Nance, 2021). Previously, we proposed that MRCK-1 functions downstream of *cdc-42* to promote endocytic recycling in the canal (Lant et al., 2015), but our new findings suggest that MRCK-1 functions independently of CDC-42 in this context. In addition to the CRIB domain, localization of MRCK-1 to the cell membrane is mediated by a tripartite module of the C1, PH and CNH domains, which bind preferentially to specific phosphatidylinositol phosphates (PIPs) (Truebestein et al., 2023). It is possible that, for excretory canal outgrowth, the C1-PH-CNH module alone is sufficient for localization of MRCK-1. In the future, a role for the C1-PH-CNH module in MRCK-1 localization should be investigated to determine whether they are required for MRCK-1 membrane binding or function.

We have shown that MRCK-1 is required for outgrowth of the excretory canal when the posterior canals follow guidance cues to extend to their target positions at the tail of the worm. Other studies of MRCK function in tubulogenesis have placed it downstream of Cdc42 signaling for lumen formation and invasion of endothelial cells in 3D culture (Koh et al., 2008; Norden et al., 2016). However, our observations in the excretory canal indicate that MRCK-1 promotes forward growth of the tube, rather than lumen formation, as *mrck-1*/MRCK-1^canal-^ mutants form a lumen despite having extremely truncated canals. Although MRCKs in other organisms are not known to regulate biological tube outgrowth, they have been shown to function in neurite outgrowth and guidance in mammalian cell culture and the *Drosophila* visual system (Chen et al., 1999; Gontang et al., 2011; Groeger and Nobes, 2007). Many of the same molecular guidance cues that direct axon outgrowth in *C. elegans* also direct excretory canal outgrowth (Hedgecock et al., 1990; Katidou et al., 2013; Marcus-Gueret et al., 2012; McShea et al., 2013; Schmidt et al., 2009; Stringham et al., 2002) and it is well established that this also holds true for guidance factors that direct the vertebrate vascular and nervous systems (reviewed by Weinstein, 2005). Therefore, we hypothesize that MRCK-1 functions in a similar manner to promote canal and axon outgrowth by activating non-muscle myosin in response to guidance cues, although the identity of these cues and how they regulate MRCK-1 remains to be elucidated. In addition to outgrowth, MRCK-1 may also function during the maintenance phase of canal development. Expression of MRCK-1 was observed in the canal throughout larval development and into adulthood, suggesting that it may be required during all these stages. In the future, inducible depletion of MRCK-1 in the canal could be carried out at later developmental stages to see whether it is also required for maintenance of its integrity.

Our data suggest that MRCK-1 functions in the canal to promote phosphorylation of the regulatory light chain (RLC) MLC-4 to activate non-muscle myosin. Although we cannot conclude that MRCK-1 directly phosphorylates MLC-4 in the canal, we have shown that the myosin light chain phosphatase *mel-11* does not appear to function downstream of *mrck-1* in this cell. Thus, it is unlikely that MRCK-1 inhibits *mel-11* to promote MLC-4 phosphorylation in the canal, but it remains to be determined whether MRCK-1 can directly phosphorylate MLC-4 in this context. It is likely that MRCK-1 has other phosphorylation targets in the canal, although the ability of the MLC-4 phosphomimetic mutant to restore canals in *mrck-1* mutants to wild-type length, and our observations that MRCK-1^canal-^ and MLC-4^canal-^ mutants have the same degree of canal truncation suggests this to be the primary mechanism through which MRCK-1 promotes canal outgrowth. The similarity of defects caused by loss of MRCK-1 and MLC-4 in the canal reinforce the notion that MRCK-1 is the sole kinase regulating activation of non-muscle myosin in this context.

Our demonstration that MLC-4/non-muscle myosin is required for canal outgrowth provides the first example of a function for non-muscle myosin in seamless tube development. Non-muscle myosin activation has been reported to function downstream of Cdc42/Rac1/Pak4 and RhoA/ROCK for lumen formation and maintenance of lumen diameter in 3D-cultured endothelial cells (Barry et al., 2016), and downstream of Rok/ROCK for salivary gland tubulogenesis in *Drosophila* (Röper, 2012). These examples of non-muscle myosin function in multicellular tubes involve the regulation of cell-cell junctions, which are absent in the seamless excretory cell. Based upon the localization of the translational MLC-4DD reporter to the growing tip of the canal, and its cell-autonomous function during canal outgrowth, it is likely that MLC-4/non-muscle myosin acts in a manner that is distinct from previously characterized examples in multicellular tubes. Instead, we hypothesize that MLC-4/non-muscle myosin function in the canal is similar to its function in neurites. Non-muscle myosin regulates the structure of neurites and their growth cones in different and sometimes oppositional manners, due to the expression of a variety of essential heavy chain isoforms and local activation by different kinases (Costa and Sousa, 2020). Rho/ROCK enhanced non-muscle myosin contraction of actin arcs promotes growth cone retraction (Zhang et al., 2003), while its activity can also promote retrograde flow and recycling of actin bundles in the growth cone to fuel axon extension (Medeiros et al., 2006). In *C. elegans* non-muscle myosin is required for dendrite self-avoidance (Sundararajan et al., 2019) and new growth cone formation for axon regeneration after injury (Shimizu et al., 2018). MRCK-driven activation of non-muscle myosin has been shown to promote neurite outgrowth in mammalian cell culture and axon guidance in the *Drosophila* visual system (Chen et al., 1999; Gontang et al., 2011). The function of non-muscle myosin activation downstream of MRCK-1 in the canal remains to be elucidated. We have shown that F-actin localization to the growing tip is not perturbed in *mrck-1* mutants, as F-actin is present throughout the long unlumenized cytoplasmic projections in these mutants. A similar defect is seen in neurite growth cones of the marine mollusk *Aplysia* after chemical inhibition of non-muscle myosin, where reduced actin retrograde flow and actin-bundle severing causes aberrant elongation of filopodia through extended F-actin bundles (Medeiros et al., 2006). Non-muscle myosin activity may be required in the canal to promote actin recycling in a similar manner, and the reduction of actin-bundle severing may be the underlying cause of the cytoplasmic projections seen upon loss of MRCK-1 or MLC-4.

In summary, we describe a new function for MRCK-1-driven activation of non-muscle myosin in seamless tube outgrowth that is independent of its canonical regulator CDC-42. Our findings show that the CRIB domain is dispensable for MRCK-1 function in the excretory canal, challenging the assumption that MRCKs require Cdc42 for proper function *in vivo*. It remains to be determined what functions upstream of MRCK-1 in the canal to regulate its activation of non-muscle myosin, and how this promotes canal outgrowth. This work deepens our knowledge of excretory canal outgrowth in *C. elegans*, which may reveal conserved mechanisms of outgrowth for seamless tubes in other organisms, such as the vertebrate microvasculature.

## MATERIALS AND METHODS

### *C. elegans* strains and maintenance

All worms were grown at 20°C on Nematode Growth Media (NGM) agar plates seeded with *Escherichia coli* OP50 bacteria (Brenner, 1974). The following strains were obtained from the *Caenorhabditis* Genetics Center (CGC), which is funded by NIH Office of Research Infrastructure Programs (P40 OK010440): FX30161, LP463, EG6699, WH556 and VC141. Strain FT1722 carrying the integrated *canalp::zif-1* transgene was generously provided by Dr Jeremy Nance (University of Wisconsin, WI, USA) (Abrams and Nance, 2021). Strain GS6603, carrying the canal-specific LifeAct::TagRFP reporter, was generously provided by Dr Daniel Shaye (University of Illinois, IL, USA) (Shaye and Greenwald, 2015). Strain BK205 carrying the canal-specific RAB-11::mCherry reporter was generously provided by Dr Matthew Buechner (University of Kansas, KA, USA) (Mattingly and Buechner, 2011). See Table S1 for all strains used in this study and their genotypes.

### CRISPR/Cas9 generation of mutant alleles and transgenes

CRISPR/Cas9 genome editing was performed by direct injection of ribonucleoprotein (RNP) complex of Cas9 protein and guide RNA(s) (gRNA), as described previously (Paix et al., 2015; Popiel and Derry, 2020). All gRNAs were composed of a universal tracrRNA from IDT and target-specific crRNA(s). For deletion and substitution edits to generate *mrck-1* null, *mrck-1ΔCRIB* and *mrck-1(on174*[K112M]*)*, single-stranded DNA (ssDNA) repair templates were ordered from Eurofins. For the deletion edit to generate the *mrck-1ΔKin* allele, and for the insertion of the ZF1 recognition motif sequence at the *mrck-1* C-terminus and *mlc-4* N-terminus, ssDNA repair templates were generated using PCR of dsDNA template followed by digestion by lambda exonuclease, as described previously (Eroglu et al., 2023). All CRISPR generated alleles were confirmed by Sanger Sequencing of PCR products by The Centre for Applied Genomics (TCAG). The full list of crRNA, repair template and primer sequences used for CRISPR/Cas9 genome editing are available in the Table S2.

### Generation and/or integration of additional transgenes

The cytosolic and apical membrane canal marker *t28h11.8p::yfp::sl2::ifb-1::cfp* (*canalp::yfp::sl2::ifb-1::cfp*) was integrated as a single copy insertion from the plasmid pJA043 (a gift from Dr Jeremy Nance) using the Mos1-mediated Single Copy Insertion (MosSCI) protocol, as previously described (Frøkjær-Jensen et al., 2008).

Extrachromosomal array onEx79 for overexpression of MLC-4(DD) was generated by microinjection of plasmid pWD285 (*exc-9p::gfp::mlc-4(T15D, S18D)::UTR_unc-54_*) at 10 ng/µl with co-injection markers pCFJ90 (Addgene plasmid 19327) and pCFJ104 (Addgene plasmid 19328) at 2.5 ng/µl and 5 ng/µl concentrations, respectively. Microinjection was performed using a FemtoJet (Eppendorf) microinjection system with an inverted Leica DMI3000B microscope.

The plasmid pWD285 was constructed by cloning the *gfp::mlc-4::UTR_unc-54_* sequence from pML1522 (a gift from Dr Michel Labouesse, Institut de Biologie Paris-Seine, France) (Gally et al., 2009) into the backbone of pBK162 (a gift from Dr Matthew Buechner), which contains the *exc-9* promoter for expression in the canal. Q5 Site-Directed Mutagenesis (New England BioLabs) was used to introduce nonsynonymous mutations into the *mlc-4* sequence to generate *mlc-4(T15D, S18D).*

### Microscopy

All microscopy was performed on live worms slide-mounted in M9 buffer with 5 µL tetramisole anesthetic (20 mM) on flat agarose pads (4%). All images show late L4 stage or early adult hermaphrodites unless otherwise specified. Representative confocal images of canal lengths, morphology and reporter constructs were captured with a Leica DMI8 (TCS SP8) lightning confocal/light sheet microscope, 20× NA 0.75 water immersion, 40× NA 1.3 water immersion (motCORR) or 63× NA 1.3 glycerol immersion objectives, 405, 448, 488, 552 and 638 nm lasers, HyD detectors, and 1× to 5× digital zoom. Leica LAS X and Lightning module software was used for image acquisition and processing. For whole-worm images, Leica LAS X ‘tile scanning’ with automatic stitching was used to capture the entire worm, and maximum intensity projections of *z*-stacks were used to show the whole canal. For MRCK-1::GFP translational reporter images, the brightness and contrast was altered for each image after acquisition using ImageJ (NIH) to improve the visibility of the GFP signal in the canal, and, as such, GFP signal should not be used to approximate MRCK-1 expression levels in the canal at different developmental stages. For F-actin and GFP::MLC-4DD localization, the same laser power and exposure times were used within experiments, and image brightness was not altered after acquisition. For analysis of canal length and morphology, fluorescent and bright-field images were viewed with a Leica DMRA2 compound microscope equipped with epifluorescence and Nomarski optics using 10× NA 0.4 or 40× NA 1.25-0.75 oil immersion objectives. Images were captured with a Hamamatsu C4742-95 digital camera using OpenLab software v5.5.2 (PerkinElmer).

### Relative excretory canal length measurements

Relative excretory canal lengths are the ratio of the length of one posterior branch of the canal to the length of the worm body. Posterior canals were measured from the cell body of the excretory canal cell to the tip of the canal. For canals with cytoplasmic projections, the canal was measured to the furthest lumenized part of the canal. One posterior canal for each individual was measured, chosen based upon clarity when focusing the microscope. If both canals were visible, the longest canal was measured. The length of the worm was measured from the excretory canal cell body to the tail of the worm, immediately past the anal pore. Measurements were made using ImageJ (NIH) software freehand trace and measurement tools. See previously published method for details (Popiel and Derry, 2020).

### Maternal embryonic/larval lethality

To quantify the maternal embryonic/larval lethality of *mrck-1* mutants and controls, L4 worms (P0) were singled out onto small NGM plates and left to lay progeny for 24 h. After the initial 24 h, P0 individuals were moved to a fresh plate each day to facilitate counting. P0 s were allowed to lay progeny (F1) for 5 days in total. After 24 h on each plate, the number of eggs laid was recorded, and the plates were monitored for 5 days each to record the number of progeny that reached adulthood (adult F1s were removed from the plate before they began to lay F2 progeny). The total number of progeny laid and the total progeny that grew to adulthood were summed across all five plates for each P0 to calculate the survival to adulthood of progeny.

### RNA interference by feeding

Knockdown of target genes by RNAi was performed by feeding *C. elegans* HT115 *E. coli* strain bacteria from the Ahringer library (Kamath and Ahringer, 2003). This library contains predicted gene sequences from the *C. elegans* genome cloned into the vector L4440 and transformed into HT115 *E. coli*. Bacterial colonies were grown overnight on an orbital shaker at 37°C in LB broth with ampicillin (final concentration 100 μg/ml) and tetracycline (final concentration 10 μg/mL). Expression of dsRNA was induced by addition of Isopropyl β-D-1-thiogalactopyranoside (IPTG) for a final concentration of 0.4 mM, and cultures remained on the orbital shaker at 37°C for an additional 4 h after induction. Cultures were concentrated 5× before being seeded onto small NGM agar plates supplemented with carbenicillin (25 μg/ml) and IPTG (2.5 mM). Bacterial lawns were dried and allowed to grow overnight at room temperature before worms were plated. L4 worms were placed on plates left for 48 h to reach adulthood and lay eggs before being removed. Knockdown of *Y95B8A_84.g*, a non-expressed pseudogene, was used as a control for RNAi experiments (Lehner et al., 2006).

### Fluorescence intensity at canal tip

The relative fluorescence intensity of GFP::MLC-4DD at the tip of the canal was quantified using ImageJ. The canal tip and an adjacent control region were selected, and a region of interest (ROI) was created to select all pixels above threshold 20 in the image selection. Mean gray value for this selection was used as a measure of fluorescence intensity to control for differences between the area of the tip and control regions. The same tip and control regions were used to measure the fluorescence intensity of the cytoplasmic RAB-11::mCherry reporter as a control.

### Statistics

Statistical analysis was performed using GraphPad Prism 9 for all tests except Fisher's exact test, which was performed using Rstudio (Posit Software, PBC). Statistical tests and significance levels (*P*-values) are specified in the figure captions. All tests performed were two-tailed. During imaging, individuals were selected based upon developmental stage and orientation on the slide for ease of imaging. No data points were excluded from analysis. For canal defects in adult worms, three or four biological replicates from *n*≥24 individuals for each genotype were pooled to calculate the percentage of the population with the phenotype (except for *mrck-1ΔKin* mutants, which had two replicates pooled for a total *n*=51). For canal defects in larval stage worms, one biological replicate of *n*≥19 individuals for each developmental stage was used. See Table S3 for details of the biological replicates and sample size for each genotype.

## Supplementary Material



10.1242/develop.202772_sup1 Supplementary information

Table S1. *C. elegans* strain names and genotypes used for this study.

Table S2. Molecular reagents and their sequences (if available) used for this study.

Table S3. Sample sizes of biological replicates for canal defect experiments.
