# Supplementary material for: MRCK-1 activates non-muscle myosin for outgrowth of a unicellular tube in Caenorhabditis elegans
Source: Development. 2024 Nov 29;151(23):dev202772. doi: 10.1242/dev.202772 (PMC11634028; doi:10.1242/dev.202772)
Supplement: Supplementary information [file develop-151-202772-s1.pdf]

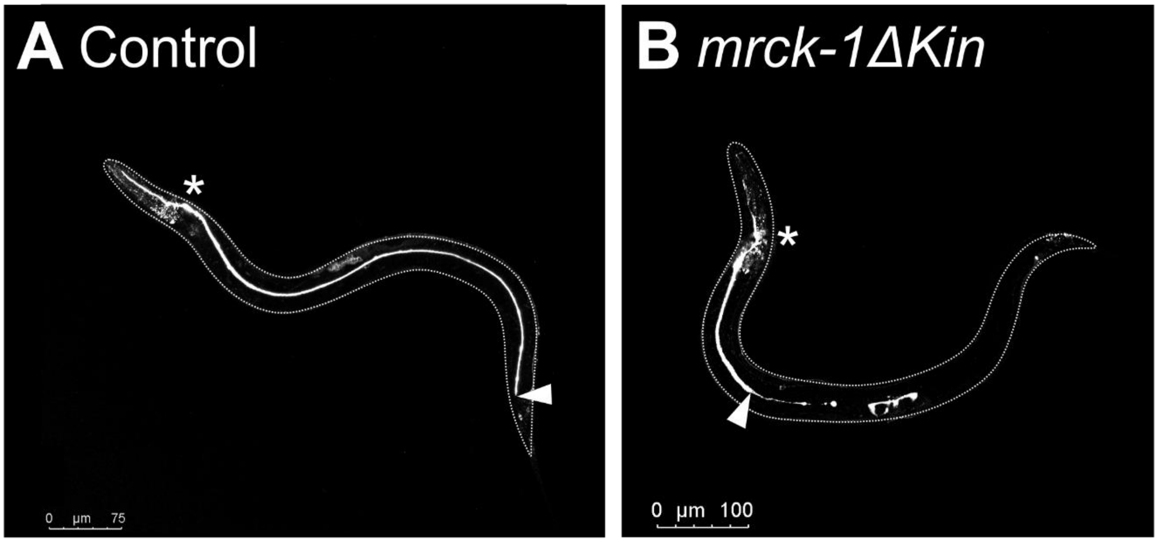

Fig. S1. *mrck-1ΔKin* mutant worms (B) have severely truncated excretory canals compared to wild-type controls (A).

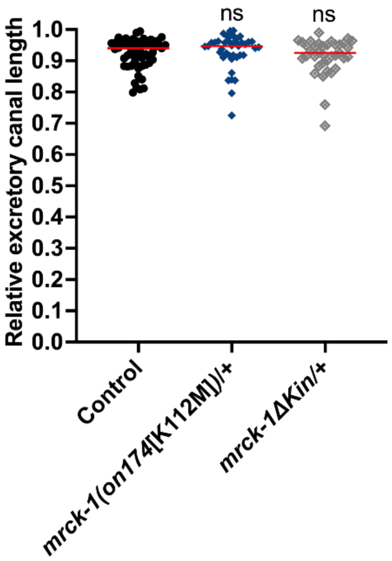

Fig. S2. *mrck-1* kinase mutant alleles do not function in a dominant-negative manner. There is no significant difference in the relative canal lengths of *mrck-1*(*on174*[K112M]) (n=40) or *mrck-1ΔKin* (n=37) heterozygotes and wild-type control worms (n=58). ns $p \geq 0.05$ (Mann-Whitney test versus wild-type control). Red lines denote population medians. Controls are wild-type worms carrying the *exc-9::mCherry::rab-11* canal marker (BK205).

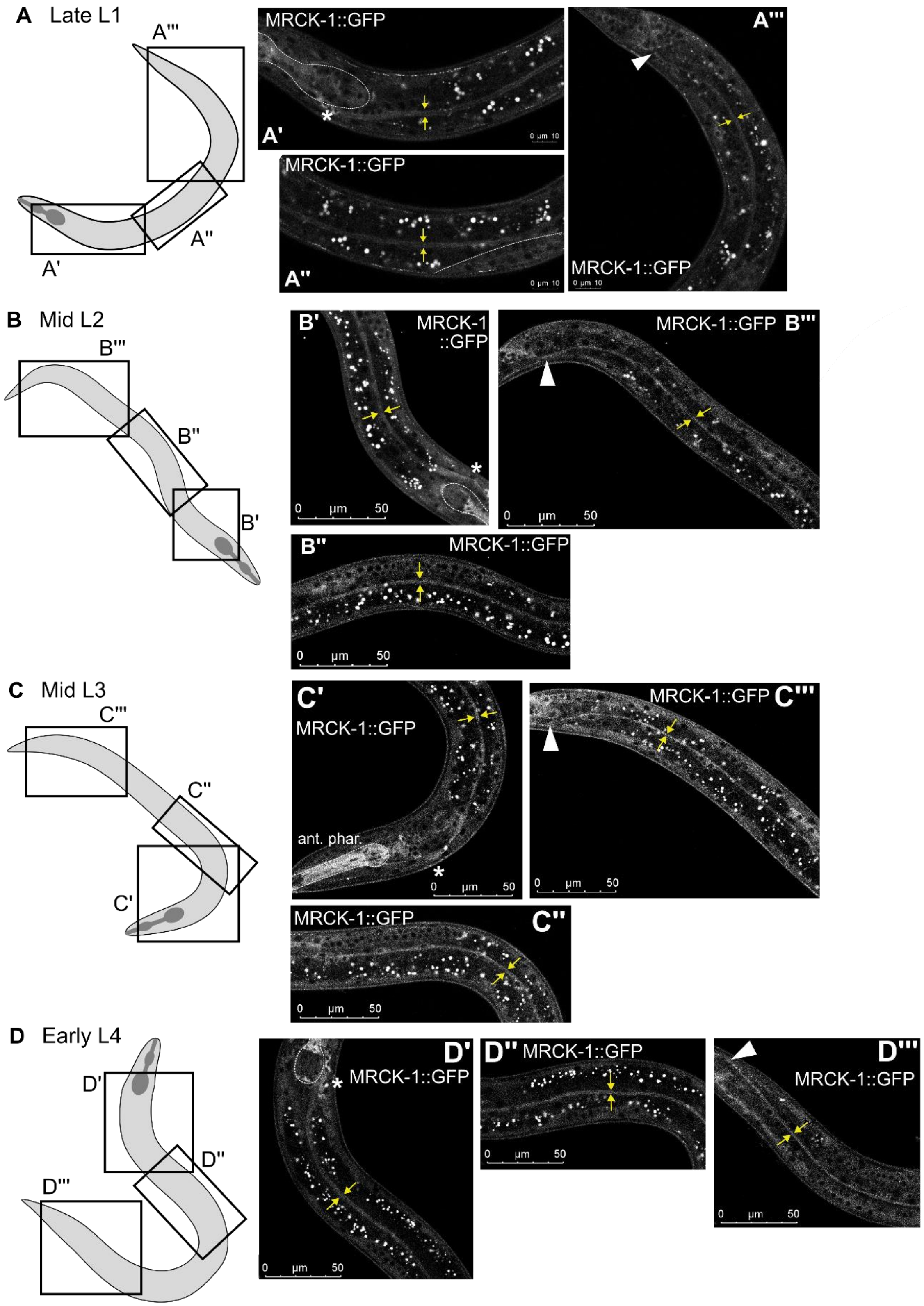

Fig. S3. MRCK-1 is expressed in the excretory canal from late L1 stage to early L4 stage. MRCK-1::GFP can be seen throughout the cytoplasm in the anterior, midbody, and posterior sections of the canal during each larval stage. Black boxes on the illustrations indicate the location of the corresponding microscopy image in the worm body plan. Asterisks denote the cell body of the canal, yellow arrows highlight the basal membranes of the canal, and the white arrowheads denote the end of the canal. MRCK-1 expression can be seen in additional tissues including the pharynx (white dotted line A', B', C', D') and the germline (white dotted line A'').

Development • Supplementary information

A *mrck-1::ZF1*

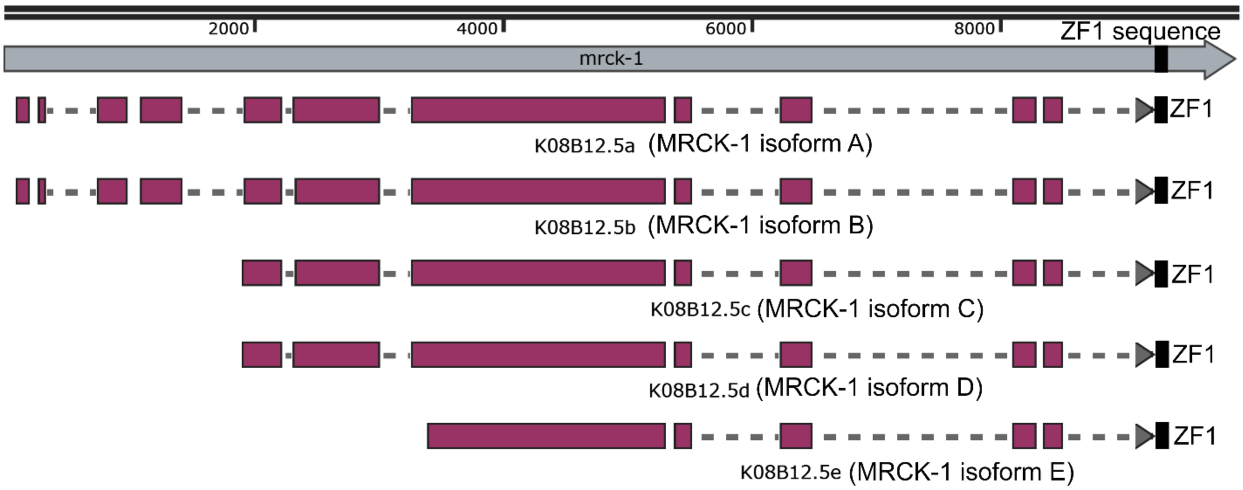

B *ZF1::mlc-4*

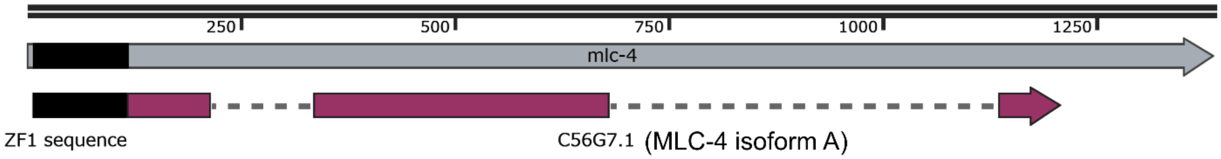

Fig. S4. *mrck-1::ZF1* and *ZF1::mlc-4* alleles add the ZF1 recognition motif to all isoforms of the target proteins. (A) The *mrck-1::ZF1* allele carried by the MRCK-1^{canal-} mutants adds the ZF1 recognition motif sequence to all 5 transcripts which encode MRCK-1 isoforms. **(B)** The *ZF1::mlc-4* allele carried by the MLC-4^{canal-} mutants adds a new start codon and ZF1 recognition motif sequence to the transcript that encodes the sole MLC-4 isoform. Gray arrows indicate gene sequence, black boxes indicate the ZF1 sequence, purple boxes indicate transcript exons, dashed lines indicate transcript introns, and the ruler indicates the relative sequence length in base pairs.

Development • Supplementary information

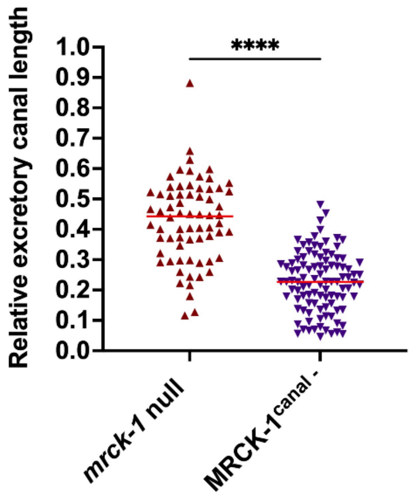

Fig. S5. Maternally contributed *mrck-1* partially suppresses canal truncations in *mrck- 1* null mutants. MRCK-1^{canal-} mutants (n=102) have significantly shorter canals than *mrck-1* null mutants (n=68) due to a loss of maternally supplied *mrck-1*. **** $p < 0.0001$ (Mann-Whitney test). Red lines denote population medians.

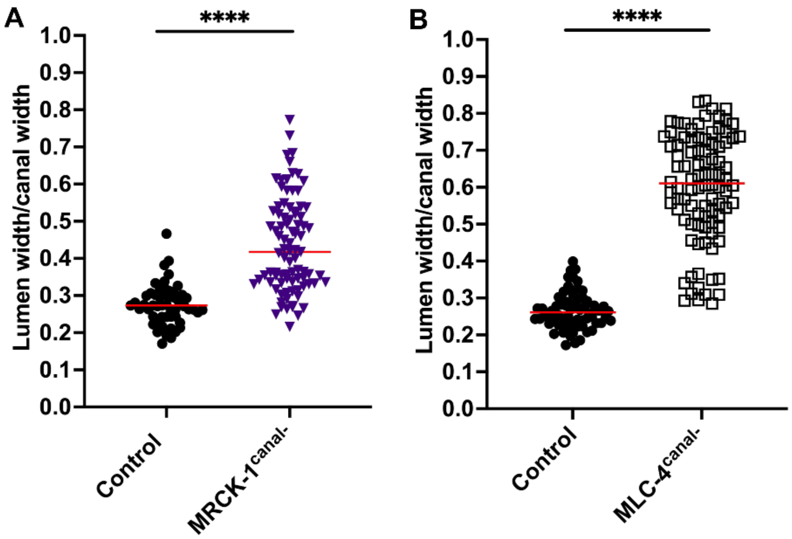

Fig. S6. MRCK-1^{canal-} and MLC-4^{canal-} mutants have canals with expanded lumens. (A) MRCK-1^{canal-} (n=87) adults have significantly higher lumen/canal width ratios compared to wild-type controls (n=55). **(B)** MLC-4^{canal-} (n=94) adults have significantly higher lumen/canal width ratios compared to wild-type controls (n=64). **** $p < 0.0001$ (Mann-Whitney test). Red lines denote population medians. **(A)** Controls are worms carrying the *t28h11.8p::yfp::sl2::ifb-1::cfp* canal marker and *t28h11.8p::zif-1* canal-specific ZIF-1 transgenes (WD982). **(B)** Controls are worms carrying the *t28h11.8p::yfp::sl2::ifb-1::cfp* canal marker, *t28h11.8p::zif-1* canal-specific ZIF-1 transgene, and *zif-1(gk117)* loss of function mutation (WD1032).

Development • Supplementary information

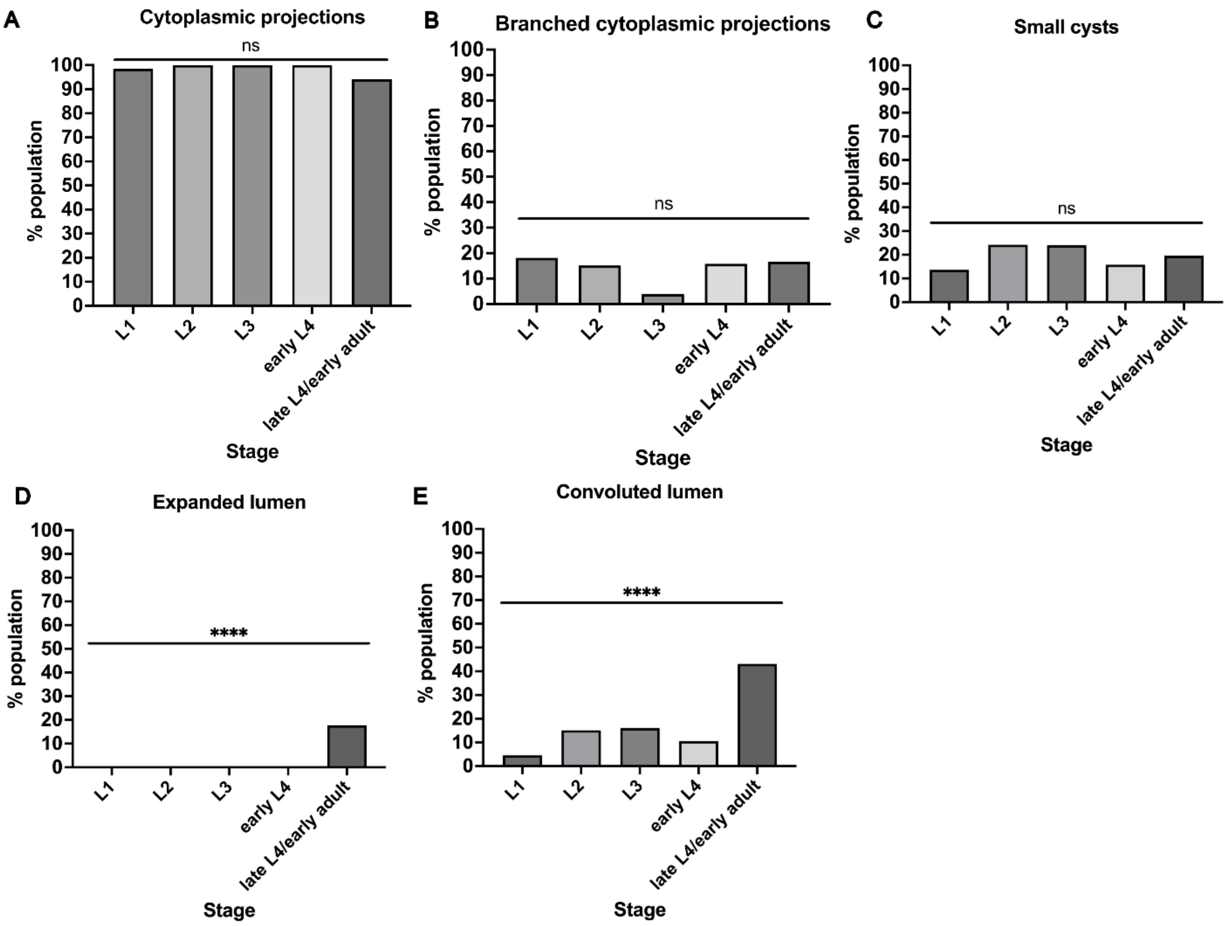

Fig. S7. Loss of MRCK-1 in the excretory cell causes cytoplasmic projections and small cysts defects during early development. (A-C) In MRCK-1^{canal-} mutants, cytoplasmic projections (single and branched) and small cysts were observed in the first larval stage and their frequency did not change significantly throughout development. **(D, E)** Expanded and convoluted lumens were not observed frequently in the population until maintenance during canal development (L2 stage or later), and their frequency significantly increased into adulthood. $n \geq 19$ (see Materials for details). ns $p \geq 0.05$, **** $p < 0.0001$ (Fisher's exact test).

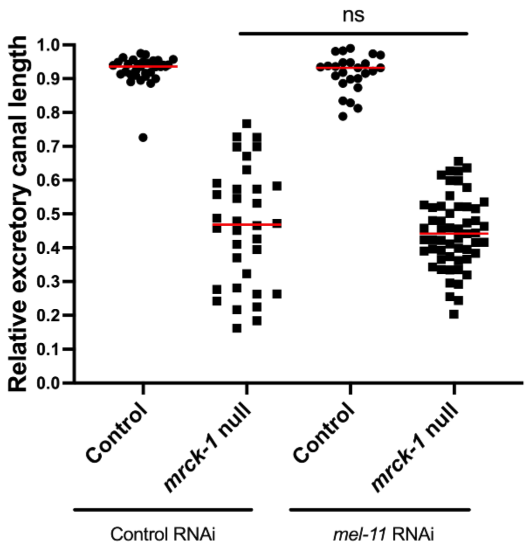

Fig. S8. Knockdown of *mel-11* does not rescue canal truncations in *mrck-1* null mutants. There is no significant difference between canal lengths of *mrck-1* null mutants treated with *mel-11* (n=55) and control (n=34) RNAi. ns $p \geq 0.05$ (Mann-Whitney test). Red lines denote population medians. Controls are wild-type worms carrying the *exc-9::mCherry::rab-11* canal marker (BK205).

Development • Supplementary information

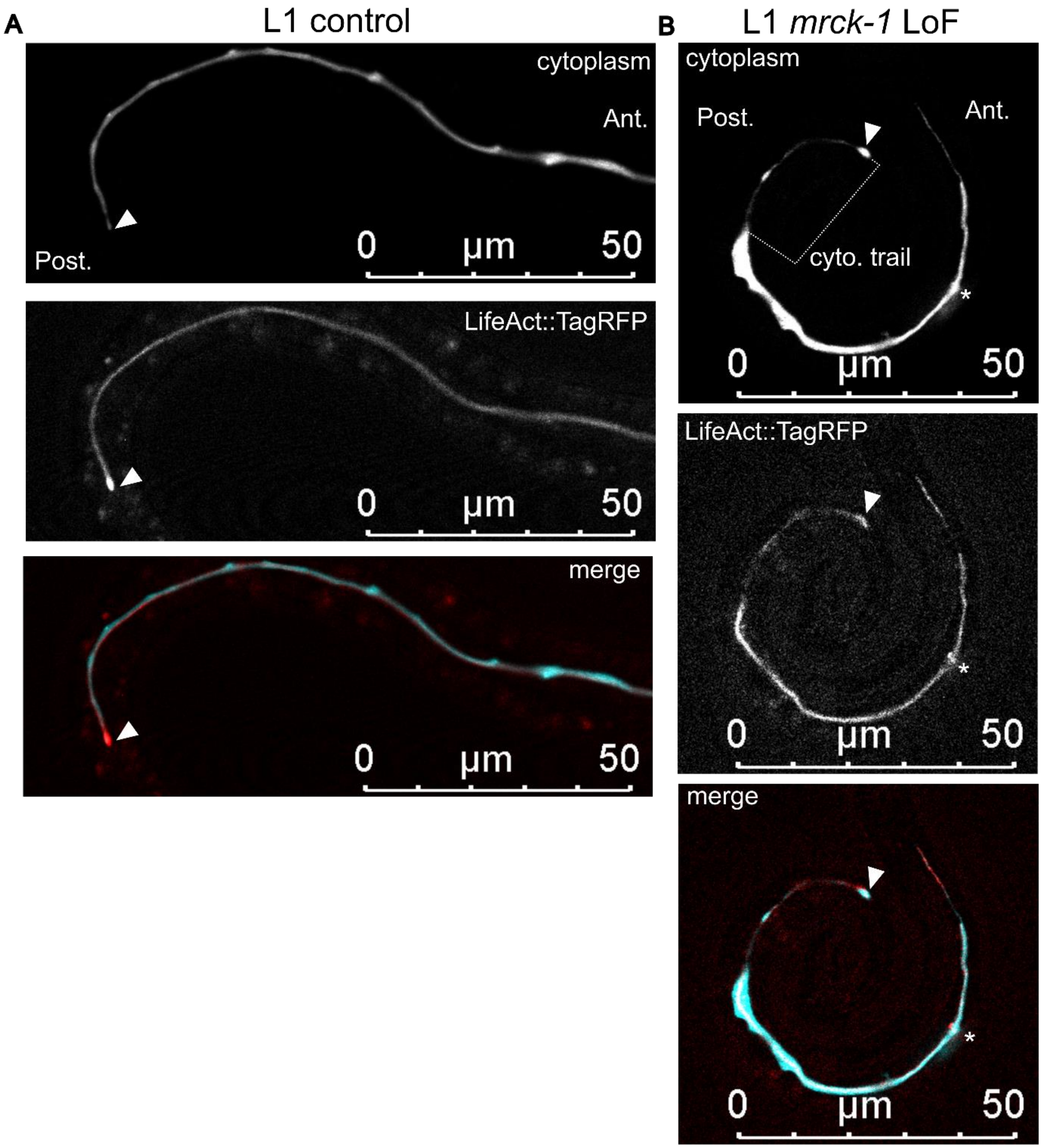

Fig. S9. Loss of *mrck-1* does not perturb F-actin localization to the growing tip of the canal during outgrowth. In both **(A)** wild-type control and **(B)** *mrck-1* LoF mutant L1 worms a marker for F-actin (LifeAct::TagRFP) is enriched at the tip of the canal (white arrowhead). Anterior (ant.) and posterior (post.) of the worms are labeled. White asterisks denote the cell body of the excretory canal (out of frame for the control). Control is a worm carrying the *glt-3p::CFP* canal marker and *glt-3p::LifeAct::TagRFP* transgene (GS6603).

Development • Supplementary information

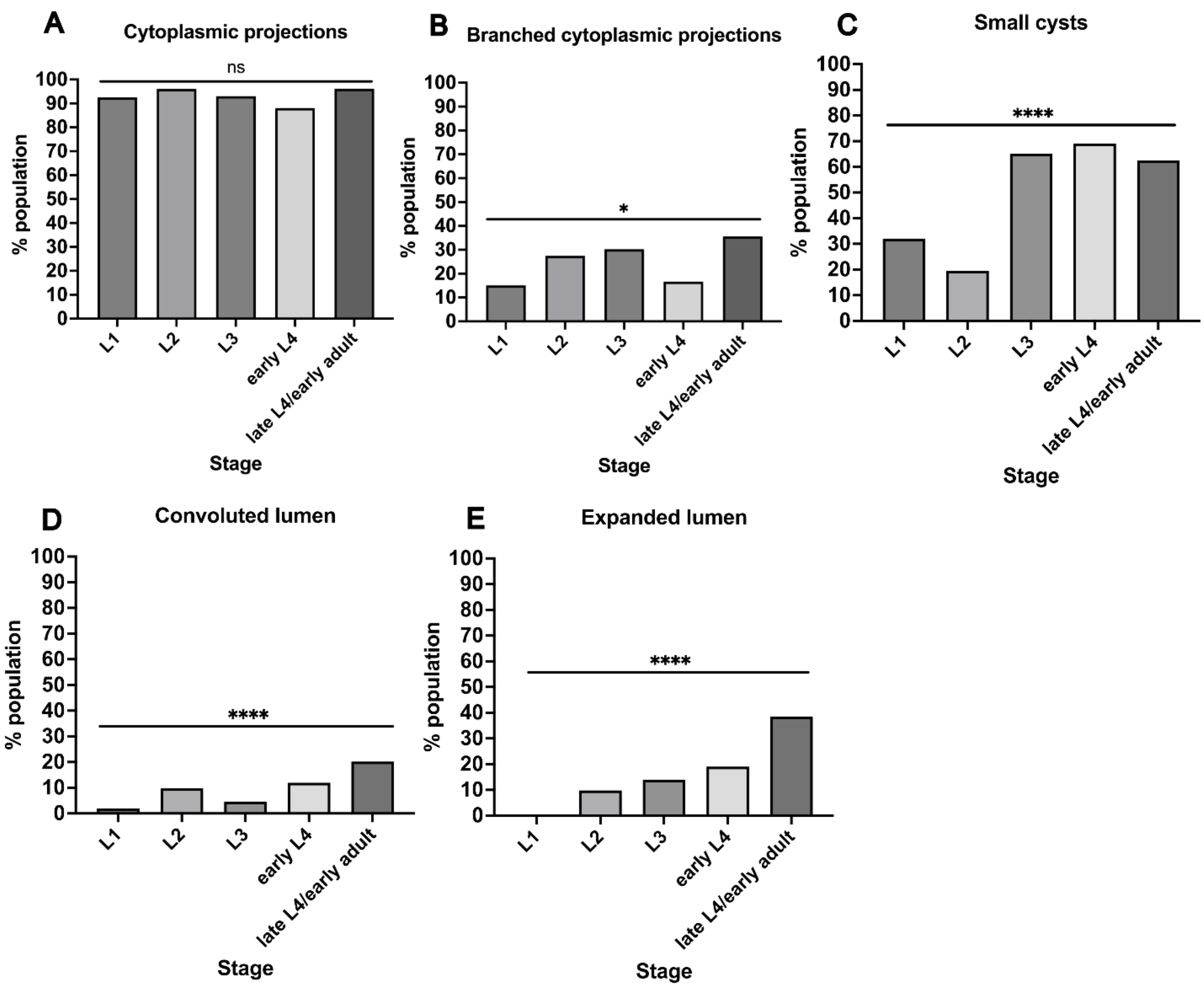

Fig. S10. Loss of MLC-4 in the excretory cell causes cytoplasmic projections and small cyst defects during outgrowth. (A-C) In MLC-4^{canal-} mutants, cytoplasmic projections (single and branched) and small cysts are present by L1 during canal outgrowth, while **(D, E)** convoluted and expanded lumens are observed during the maintenance phase (L2 or later). **(A)** The prevalence of cytoplasmic projections does not change during development, while **(C-E)** the prevalence of small cysts, convoluted lumens, and expanded lumens increases with age. n ≥ 36 (see Materials for details). ns $p \geq 0.05$, * $p < 0.05$, **** $p < 0.0001$ (Fisher's exact test).

Development • Supplementary information

Table S1. *C. elegans* strain names and genotypes used for this study.

Available for download at
https://journals.biologists.com/dev/article-lookup/doi/10.1242/dev.202772#supplementary-data

Table S2. Molecular reagents and their sequences (if available) used for this study.

Available for download at
https://journals.biologists.com/dev/article-lookup/doi/10.1242/dev.202772#supplementary-data

Table S3. Sample sizes of biological replicates for canal defect experiments.

Available for download at
https://journals.biologists.com/dev/article-lookup/doi/10.1242/dev.202772#supplementary-data
