# Supplementary material for: MRCK-1 activates non-muscle myosin for outgrowth of a unicellular tube in Caenorhabditis elegans
Source: Development. 2024 Nov 29;151(23):dev202772. doi: 10.1242/dev.202772 (PMC11634028; doi:10.1242/dev.202772)
Supplement: Supplementary file 1 [file develop-151-202772_review_history.pdf]

MRCK-1 activates non-muscle myosin for outgrowth of a unicellular tube in *Caenorhabditis elegans*

Evelyn M Popiel, Rhea Ahluwalia, Stefan Scheutz, Bin Yu and William Brent Derry
DOI: 10.1242/dev.202772

Editor: Swathi Arur

Review timeline
Original submission: 6 February 2024
Editorial decision: 27 March 2024
First revision received: 23 September 2024
Editorial decision: 23 October 2024
Second revision received: 24 October 2024
Accepted: 28 October 2024

Original submission

First decision letter

MS ID#: DEVELOP/2024/202772

MS TITLE: MRCK-1 activates non-muscle myosin for outgrowth of a unicellular tube in *Caenorhabditis elegans*

AUTHORS: Evelyn M Popiel and William Brent Derry

I have now received all the referees' reports on the above manuscript, and have reached a decision. The referees' comments are appended below, or you can access them online: please go to BenchPress and click on the 'Manuscripts with Decisions' queue in the Author Area.

As you will see, the referees find the work well controlled and well conducted, providing insights into the seamless tube biology, although there is some disagreement between the reviewers on the level of mechanism provided. After much thinking about the recommendations from reviewers, I believe that incorporating the suggestions from reviewers 2 and 3 will further improve the clarity and rigor of the analysis and be useful for the field. If you are able to revise the manuscript along the lines suggested, which may involve further experiments, I will be happy receive a revised version of the manuscript. Your revised paper will be re-reviewed by one or more of the original referees. Thus, please attend to all of the reviewers' comments and ensure that you clearly highlight all changes made in the revised manuscript. Please avoid using 'Tracked changes' in Word files as these are lost in PDF conversion. I should be grateful if you would also provide a point-by-point response detailing how you have dealt with the points raised by the reviewers in the 'Response to Reviewers' box. If you do not agree with any of their criticisms or suggestions please explain clearly why this is so.

Reviewer 1

Advance summary and potential significance to field

The authors characterize the function of the worm Myotonic Dystrophy-Related Kinase homolog MRCK-1 during outgrowth of the excretory canal, a seamless tube running along the length of the

© 2024. Published by The Company of Biologists under the terms of the Creative Commons Attribution License (https://creativecommons.org/licenses/by/4.0/).

animal. Using CRISPR technology, they establish that MRCK-1 is required throughout development for canal outgrowth, that its CDC42-interacting domain is not required but that its kinase domain is required. They further show that MRCK-1 is likely to act through one of its well-known targets, the non-muscle regulatory light chain MLC-4, since excretory canal expression of a phosphomimetic MLC-4 form can rescue a MRCK-1 null allele. Furthermore, in the absence of MLC-4 or MRCK-1, the canal does not extend, although it can still make a long projection. MLC-4 being located at the canal tip, it indicates that it is not simply required for extension of that projection.

Comments for the author

The work is very well done and the results are convincing, in particular because it relies on CRISPR knockins. However, it is conceptually not so novel since MRCK-1 and MLC-4 are known to work together. The only novel finding is that MLC-4 is somehow required in the excretory canal. The authors unfortunately could not localize MRCK-1 in the canal for technical reasons, did not link MRCK-1 to other players acting in excretory canal outgrowth, and could not elucidate the precise function of MLC-4. They speculate that MLC-4 could be required to mediate actin retrograde flow, as found in Aplysia axons. Altogether, I do not recommend publication in Development.

Reviewer 2

Advance summary and potential significance to field

In this manuscript, Popiel and Derry investigate the role of the conserved kinase, MRCK-1, in the outgrowth of the excretory canal (EC), a seamless tube in C. elegans. The authors find that relative to controls, the excretory canal was significantly shorter in ok586 mrck-1 loss-of-function mutants, mrck-1 deletion mutants, and in mrck-1 KD mutants (which contain a single point mutation predicted to eliminate kinase activity). The authors found that the EC was shorter during both outgrowth and maintenance phases (from the first larval stage, L1, through adult stages). The authors additionally found that EC outgrowth was perturbed by degradation of MRCK-1 specifically from the EC, suggesting MRCK-1 acts in a cell autonomous manner. In addition to the shorter length of ECs, the authors found multiple defects in the structure of the EC in mrck-1 mutants, including “cytoplasmic projections” which were un-lumenized regions of the EC, convoluted lumens, expanded lumens, and small cysts within the EC cytoplasm. Deletion of the CRIB domain (which mediates the interaction between MRCK-1 and CDC-42) resulted in ECs that were significantly shorter in mrck-1ΔCRIB mutant L4/early adult worms than in control worms but had far less of an effect on the EC than the other mrck-1 mutations, suggesting that MRCK-1 primarily mediates EC outgrowth independently of its interaction with CDC-42. Given the similarity in phenotypes between the mrck-1 KD mutants and the mrck-1 loss-of-function and null mutants, the authors hypothesized that MRCK-1 drives EC elongation via the kinase domain. Although the authors do not specifically demonstrate the absence of kinase activity in their mrck-1 KD point mutants, they report that expression of a phosphomimetic form of the downstream MRCK-1 target, MLC-1 (the regulatory light chain of non-muscle myosin), rescues the EC elongation defects in the mrck-1 loss-of-function mutants.
They also demonstrate that EC specific depletion of MLC-1 phenocopies the EC outgrowth defect observed in worms depleted of MRCK-1. Overall, this work is well done and provides evidence that the conserved kinase, MRCK-1 may regulate seamless tube outgrowth through direct or indirect phosphorylation of the regulatory light chain of non-muscle myosin independently of the association of MRCK-1 with CDC-42.

This paper describes a novel role for the conserved MRCK-1 kinase and non-muscle myosin in seamless tube outgrowth. Interestingly, MRCK-1 appears to promote EC elongation primarily via its kinase activity and independently of its interaction with CDC-42, which differs from multicellular tissues that form junctions. It seems possible that the roles described here are conserved in the outgrowth of other seamless tubes in other organisms.

Comments for the author

Major comments

© 2024. Published by The Company of Biologists under the terms of the Creative Commons Attribution License (https://creativecommons.org/licenses/by/4.0/).

1) While I think it is likely that the point mutation that results in a K112M substitution results in a kinase dead version of MRCK-1, there is no validation that this mutation results in loss of kinase activity. I think using an anti-phosphorylated regulatory myosin light chain antibody as was used by Marston et al. 2016 to validate a loss of phosphorylation of MLC-4 in the excretory canals of the existing KD MRCK-1 mutants would provide stronger evidence that the kinase activity is specifically abrogated. Such antibody staining would also provide additional evidence that MRCK-1 regulates phosphorylation of MLC-4. I appreciate the difficulty in validating complete loss of kinase activity in vivo, so alternatively would like to see a second presumptive kinase dead mutant phenocopy the shorter ECs that were observed in the existing KD mutant.

2) While it seems likely that MRCK-1 does not require CDC-42 for its function in EC outgrowth, I am concerned that the small, but significant outgrowth defect reported in the late L4/early adult mrck-1ΔCRIB mutants in Figure 1 may be less severe due to maternal contribution of wild-type MRCK-1. The authors nicely demonstrate that worms from mrck-1ΔCRIB mutant mothers survive to adulthood in Figure 3, but given the significant difference in excretory canal lengths between zygotic and maternal/zygotic mrck-1 KD mutant worms, I think it is important to verify that the EC length defects aren't significantly worse in mrck-1ΔCRIB maternal/zygotic mutants compared to mrck-1ΔCRIB zygotic mutant worms.

Minor comments

1) Ideally, the authors would include localization data of MRCK-1 in the EC during outgrowth and maintenance phases, and would describe the localization of the MRCK-1 ΔCRIB and KD mutant proteins. I appreciate that the authors highlight the technical difficulties they encountered with attempting to visualize MRCK-1 in the EC in their discussion. I wonder if antibody staining against the fluorophore (or MRCK-1 directly) might allow for some description of MRCK-1 localization? Alternatively, could the authors overexpress the canal specific translational reporter in the mrck-1 null or LoF mutants to both rescue the outgrowth defects and determine MRCK-1 localization in the EC without producing the defects observed when untagged MRCK-1 is also present?

2) The CRIB domain appears to be dispensable for MRCK-1 function in EC outgrowth suggesting that MRCK-1 may not function downstream of CDC-42 as originally predicted. This is different than in some other tissues. To better understand the genetic pathway in the EC, I think it would be useful to determine if the MLC-4 phosphomimetic that rescues the MRCK-1 LoF EC elongation defects fails to rescue the EC elongation defects in CDC-42EC- worms.

3) In Figure 2C, it appears that the mutant EC lengths do not scale with the increased size of the worms as they grow from L1 to L3 to L4. Does this suggest that MRCK-1 is required for both outgrowth and maintenance phases?

4) In figure 5C, the phosphomimetic MLC-4 is shown at the tip of the growing EC during the outgrowth phase. Is the phosphomimetic form of MLC-4 also present in the EC during maintenance phases?

5) Figure 1B – add a bar similar to ok586 to represent the null mutant that was made with CRISPR.

6) Figure 1H – the unlumenized cytoplasmic projection was a little hard to see. Consider adding a higher magnification inset that shows the transition from the lumenized portion to the cytoplasmic projection.

7) Figure 2A – consider showing the EC cell the same way as portrayed in Fig. 1 for consistency and to help the readers less familiar with C. elegans physiology.

8) Figure 3 – Be sure to include sample sizes in the legend.

9) Figure 4 – The images in D-L were a bit too small to see the details described in the text. Consider making the images larger (or including enlarged insets). It would also help to add arrows like those included on Figure 1H to see both the single (4D) and branched (4F) cytoplasmic projections.

© 2024. Published by The Company of Biologists under the terms of the Creative Commons Attribution License (https://creativecommons.org/licenses/by/4.0/).

10) Figure 5A – The EC was hard to see in the mrck-1 LoF: mlc-4DD image, due to fluorescence outside of the canal. Consider using a different image, or explain the signal outside of the EC that is absent from the control.

11) Figure 5B – is the mrck-1 LoF: mlc-4DD EC length significantly different from controls?

12) Consider adding a supplemental figure to show where the ZF tags were added to mrck-1 and mlc-4 and to clarify that all isoforms (particularly of mrck-1) were tagged using this method.

13) I think the data shown in figure 7 would fit better if incorporated into figure 6, in a parallel format to the data shown in figure 4. A summary model could be included as a new figure 7.

14) Consider clarifying that the initial characterization of EC length was made in L4/adult worms in figure 1 in the text description on page 6 and/or clarifying the first sentence under the "mrck-1 is required for canal outgrowth" section on page 7 to help readers more easily follow that figure 2 is reporting on different worm stages than are reported on in figure 1.

15) On page 9, should also cite Sallee et al., 2018 for developing tissue specific degradation using the ZF/ZIF-1 system.

16) At the beginning of page 10, "the expanded lumen phenotype describes canals in which most of the excretory canal volume is taken up by lumen." It would be helpful to quantify the width of expanded lumens relative to controls using the existing data.

17) Check the manuscript for small grammatical errors. (E.g. on page 8 "it's" should be "it is", page 12 "…reporter showed enrichment growing tip…" should be "…enrichment in the growing tip").

Reviewer 3

Advance summary and potential significance to field

The well-written manuscript by Popiel and Derry examines the growth of the *C. elegans* excretory canal cell, an established model for understanding the formation and growth of single-celled tubes including mammalian capillaries. A key technique used by the investigators is degradation of proteins solely within the excretory canal cell. Through use of this technique, the authors convincingly show that: 1) Two proteins required for activation of myosin heavy chain, MRCK-1 (Myotonic dystrophy-Related, CDC42-binding Kinase homologue) and MLC-4 (Myosin Light Chain), have necessary functions in stimulating these hollow tubes to grow relative to the length of the animal in the first larval stage; 2) MLC-4 acts downstream of MRCK-1, and; 3) Surprisingly, the CDC-42-interacting domain of MRCK-1 is not required for this growth. This last is surprising since others have shown this "CRIB" domain to be necessary for generating force that increases cortical tension.

The background is nicely written and sets up quite nicely the information needed to understand the work. The advance in knowledge is solid and should interest researchers working on the functions of myosin in a range of contexts, as well as researchers studying single-celled tubular structures, from pollen tubes to endoplasmic reticula to blood capillaries. The topic is clearly relevant to the readership of *Development*. The significance of the work lies in providing a finer understanding of how myosin is activated to organize actin filaments into dynamic shapes to allow a tubular structure to grow.

The statistical methods used are valid for determining which factors are most relevant to tubule formation. The statistical graphs present all the data points and line to indicate the median of points for each set. I would like to see the data presented to include 2 more lines for each set to indicate variance above and below the median, (e.g. the boundaries of the central 65% of points), as it is not possible to see all the individual points in all of the samples.

Based on the above, I believe that this paper is suitable for *Development*. If the paper needs to be shortened, I would suggest Fig. 3 as good science, but a minor point that could be placed in the

© 2024. Published by The Company of Biologists under the terms of the Creative Commons Attribution License (https://creativecommons.org/licenses/by/4.0/).

Supplementary Data. All the other figures are clearly essential to the point being made. The figures currently in Supplementary Data are all quite relevant.

Comments for the author

Fig. 2A: I suggest drawing the worms (but not the egg) to scale, to show the dramatic difference in canal length that occurs during maintenance, similar to the growth of neurons in mammals.

In Fig. 2B: As the points for the control are identical for panels B, C, and D, these 3 graphs could be condensed if necessary, to a single graph with the dots of different forms of mrck-1 distinguished by the color and shape.
e.g. Control Null KD &[Delta]CRIB Control Null KD &[Delta]CRIB ….
L1 L2... similarly for L3, L4, adult

Fig. 4a: What is the control strain? Is it simply a wild-type worm? Or is it a worm expressing the ZIF-1 protein in the canal but not having the ZF1 recognition motif attached to the mrck-1 gene. The latter would be a better control to ensure that canal-expressed ZIF-1 does not affect canal growth or morphology.

Very minor suggestions:
The grammarian says: "Wild type" is correct when "type" is a noun, but when "type" is modifying another word, the correct form is hyphenated, e.g. "wild-type worm" or "wild-type protein." (Similarly for "dominant negative" vs. "dominant-negative mutation" or "dominant-negative fashion."

Readers may confuse "KD mutants" with "KnockDown"; consider another abbreviation such as "KinD" for "Kinase-Dead" mutants

p.12 line 3: change "showed enrichment growing tip" to "showed enrichment **at** the growing tip"

p. 13, last paragraph of the Results section, 8th line: change "…similar to the development expanded and convoluted…" to "…similar to the **of** expanded and convoluted…"

For clarity, put a page break after each figure legend, so that., e.g. Figure Legend 6 is not juxtaposed next to Figure 7, and Supp. Figure Legend S3 is not juxtaposed to Supp. Figure S4.

Supp. Fig. S4 should have asterisks to denote the cell body in the L1 control figures.

First revision

Author response to reviewers' comments

We would like to thank the reviewers for their thoughtful and insightful comments on our manuscript. It was a pleasure to receive fair reviews and useful experimental suggestions to help us to improve the quality of this work. We believe that the revised version of the manuscript addresses all concerns raised. Below are our detailed responses (*in blue*), to the specific comments and suggestions:
Reviewer 2 Comments for the Author:

Major comments

1) While I think it is likely that the point mutation that results in a K112M substitution results in a kinase dead version of MRCK-1, there is no validation that this mutation results in loss of kinase activity. I think using an anti-phosphorylated regulatory myosin light chain antibody as was used by Marston et al. 2016 to validate a loss of phosphorylation of MLC-4 in the excretory canals of the existing KD MRCK-1 mutants would provide stronger evidence that the kinase activity is specifically abrogated. Such

© 2024. Published by The Company of Biologists under the terms of the Creative Commons Attribution License (https://creativecommons.org/licenses/by/4.0/).

antibody staining would also provide additional evidence that MRCK-1 regulates phosphorylation of MLC-4. I appreciate the difficulty in validating complete loss of kinase activity in vivo, so alternatively would like to see a second presumptive kinase dead mutant phenocopy the shorter ECs that were observed in the existing KD mutant.

To further validate our 'kinase dead' allele (*mrck-1[K112M]*), we generated a "kinase domain deletion allele (*mrck-1ΔKin*) which encodes an in-frame 1350 bp deletion that removes the entire kinase domain of MRCK-1" (p.6). We found that *mrck-1ΔKin* mutants had the following phenotypes that were similar to *mrck-1[K112M]* mutants: canal defects (cytoplasmic trailing and convoluted lumen) (Fig 1 K, L), survival to adulthood of maternal zygotic mutants (MZ) (Fig 2 C), and canal lengths of MZ mutants (Fig 2 D). Notably, the canals of the *mrck-1ΔKin* zygotic (Z) mutants were significantly shorter than the *mrck- 1[K112M]* Z mutants (Fig 2A). As we postulate in the revised manuscript, "These findings also suggest that maternally supplied wild-type MRCK-1 partially suppresses canal truncations in *mrck-1[K112M]* Z mutants, but not the *mrck-1ΔKin* Z mutants. Both the alleles are predicted to encode proteins that contain the capped helix bundle (CHB) domain required for MRCK-1 dimerization, so it is possible that the MRCK-1ΔKin protein creates non-functional dimers with the maternally contributed wild-type MRCK-1, while the MRCK-1[K112M]/WT MRCK-1 dimers retain some function (Figure 2 E)." (p. 8) Due to the phenotypic similarities of the *mrck-1ΔKin* and *mrck-1[K112M]* mutants, particularly the canal lengths of MZ mutants (which are not significantly different) we believe that the K112M substitution results in a kinase dead version of MRCK-1. Since we did not test the activity of MRCK-1(K112M) by kinase assay, we have also renamed the allele *mrck-1[K112M]* in the manuscript (instead of 'kinase dead').

2) While it seems likely that MRCK-1 does not require CDC-42 for its function in EC outgrowth, I am concerned that the small, but significant outgrowth defect reported in the late L4/early adult mrck-1ΔCRIB mutants in Figure 1 may be less severe due to maternal contribution of wild-type MRCK-1. The authors nicely demonstrate that worms from mrck-1ΔCRIB mutant mothers survive to adulthood in Figure 3, but given the significant difference in excretory canal lengths between zygotic and maternal/zygotic mrck-1 KD mutant worms, I think it is important to verify that the EC length defects aren't significantly worse in mrck-1ΔCRIB maternal/zygotic mutants compared to mrck-1ΔCRIB zygotic mutant worms.

Since MZ *mrck-1ΔCRIB* mutants have wild-type levels of survival to adulthood (Fig 2 C), we maintained this strain as homozygotes. Therefore, the adults analyzed in Figure 1 are MZ mutants. We have added this clarification in the manuscript "All the previously analyzed *mrck-1* mutants were Z mutants, apart from the *mrck-1ΔCRIB* mutants which were maintained as homozygotes due to their wild-type level of survival to adulthood (Figure 2 C)." (p 8).

Minor comments

1) Ideally, the authors would include localization data of MRCK-1 in the EC during outgrowth and maintenance phases, and would describe the localization of the MRCK-1 ΔCRIB and KD mutant proteins. I appreciate that the authors highlight the technical difficulties they encountered with attempting to visualize MRCK-1 in the EC in their discussion. I wonder if antibody staining against the fluorophore (or MRCK-1 directly) might allow for some description of MRCK-1 localization? Alternatively, could the authors overexpress the canal specific translational reporter in the mrck-1 null or LoF mutants to both rescue the outgrowth defects and determine MRCK-1 localization in the EC without producing the defects observed when untagged MRCK-1 is also present?

Using a Leica SP8 confocal microscope with the lightning deconvolution module we were able to visualize a translational reporter of MRCK-1 in the canal of adults and during each larval stage, where it is expressed in the cytoplasm throughout the entire length of the canal. We added this data to the manuscript. "Using a previously published translational reporter of MRCK-1 we were able to confirm its expression in the excretory canal of adult worms (Figure 1 B-B''). We observed expression of MRCK-1::GFP in the cytoplasm of the canal through its entire length (Figure 1 B-B"). MRCK-1::GFP expression was seen in additional tissues, including the pharynx (Figure 1 B)." (p. 5) "Using the same translational

© 2024. Published by The Company of Biologists under the terms of the Creative Commons Attribution License (https://creativecommons.org/licenses/by/4.0/).

reporter as before we were able to confirm expression of MRCK-1 in the canal from late L1 stage to early L4 stage (Figure 2 B-E). MRCK-1::GFP can be seen throughout the cytoplasm in the anterior, midbody, and posterior sections of the canal during each larval stage (Figure S3)." (p. 9) "Leica LAS X and Lightning module software was used for image acquisition and processing was used for deconvolution" (p. 17)

2) The CRIB domain appears to be dispensable for MRCK-1 function in EC outgrowth suggesting that MRCK-1 may not function downstream of CDC-42 as originally predicted. This is different than in some other tissues. To better understand the genetic pathway in the EC, I think it would be useful to determine if the MLC-4 phosphomimetic that rescues the MRCK-1 LoF EC elongation defects fails to rescue the EC elongation defects in CDC-42EC- worms.

We obtained strain FT1736 (*ZF1::yfp::cdc-42)* II; *xnIs548 (canalp::zif-1)* unmapped from the Nance lab. We were able to generate an extrachromosomal array to express MLC-4DD in these mutants by injection, but we were unable to generate the control strain for the experiment (canal marker in the CDC-42^{canal-} worms). We tried to cross one of our integrated canal markers on chromosome V into the CDC-42^{canal-} strain, but failed to find any marker +ve F2s homozygous for the *canalp::zif-1* transgene (we believe this transgene may be integrated on Chr V). We attempted to introduce a canal marker by injection of pJA043 (*canalp::cfp::sl2::yfp*) plus coinjection markers. We have successfully injected this plasmid before to generate 3 different extrachromosomal arrays, but after injection of >100 hermaphrodites performed by 3 different lab members we were unable to generate a stable extrachromosomal array. It seems that completing this experiment may require more extensive troubleshooting or generation of a new CDC-42^{canal-} strain.

3) In Figure 2C, it appears that the mutant EC lengths do not scale with the increased size of the worms as they grow from L1 to L3 to L4. Does this suggest that MRCK-1 is required for both outgrowth and maintenance phases?

We expressed the canal lengths as a ratio to the total length of the worm (relative canal length), so if MRCK-1 is required during maintenance phase in the canal we would expect to see this value decrease throughout development. While there is no significant decrease in the relative canal lengths for the *mrck-1* null mutants ($p > 0.05$, Kruskal Wallis Test (non-parametric equivalent of one-way ANOVA), there is a significant decrease in the *mrck-1[K112M]* mutants ($p < 0.05$, Kruskal Wallis Test) (see below). Additionally, our findings that MRCK-1 is expressed in the canal from L1 stage to adulthood suggests that it may be required for maintenance in addition to outgrowth. The best way to directly address this question would be to deplete *mrck-1*/MRCK-1 during later stages of development using RNAi or an inducible protein degradation system. We were not able to effectively knockdown expression of *mrck-1* by RNAi, and creating an inducible system is beyond the scope of this study, although we believe it could be a fruitful direction for future research. We have added the following to our discussion to reflect this "Expression of MRCK-1 is maintained in the canal throughout larval development into adulthood, which suggests that it may be required during all these stages. In the future inducible depletion of MRCK-1 in the canal could be used during later developmental stages to see if it is also required for maintenance of the canal." (p. 16)

© 2024. Published by The Company of Biologists under the terms of the Creative Commons Attribution License (https://creativecommons.org/licenses/by/4.0/).

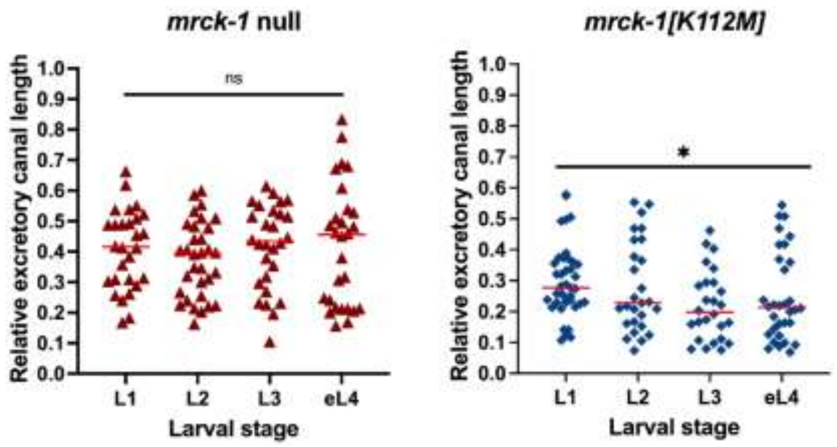

4) In figure 5C, the phosphomimetic MLC-4 is shown at the tip of the growing EC during the outgrowth phase. Is the phosphomimetic form of MLC-4 also present in the EC during maintenance phases?

The phosphomimetic MLC-4 transgene used in our experiments is a multicopy extrachromosomal array expressed under the *exc-9* promoter (strong canal promoter), so it is expressed in the EC during all stages of development. However, since this is not the endogenous promoter, we can't use this transgene to make any conclusions about the expression pattern of *mlc-4* during the canal maintenance phase.

5) Figure 1B – add a bar similar to ok586 to represent the null mutant that was made with CRISPR.

Added to manuscript Figure 1 C

6) Figure 1H – the unlumenized cytoplasmic projection was a little hard to see. Consider adding a higher magnification inset that shows the transition from the lumenized portion to the cytoplasmic projection.

Higher magnification inset added to representative image of cytoplasmic projection (Figure 1 I).

7) Figure 2A – consider showing the EC cell the same way as portrayed in Fig. 1 for consistency and to help the readers less familiar with C. elegans physiology.

Figure 2 A illustrations were updated so both the left and right canals are shown.

8) Figure 3 – Be sure to include sample sizes in the legend.

Sample sizes added to legend.

9) Figure 4 – The images in D-L were a bit too small to see the details described in the text. Consider making the images larger (or including enlarged insets). It would also help to add arrows like those included on Figure 1H to see both the single (4D) and branched (4F) cytoplasmic projections.

All representative images of canal defects in Figure 6 have been enlarged and additional arrows/arrowheads have been added to highlight the specific defects.

10) Figure 5A – The EC was hard to see in the mrck-1 LoF: mlc-4DD image, due to fluorescence outside of the canal. Consider using a different image, or explain the signal outside of the EC that is absent from the control.

We believe that the GFP signal in our *mrck-1* LoF; *mlc-4DD* worms is an artifact of the multicopy extrachromosomal array (*exc-9p::gfp::mlc-4[DD]*). The wild-type 'control' used in

© 2024. Published by The Company of Biologists under the terms of the Creative Commons Attribution License (https://creativecommons.org/licenses/by/4.0/).

this experiment has the same GFP signal, as do other strains we have made with this plasmid (*exc-9p::gfp::mlc-4[DD]*, pWD285).

11) Figure 5B – is the mrck-1 LoF: mlc-4DD EC length significantly different from controls?

Yes, the *mrck-1; MLC-4DD* mutants have canals that are significantly shorter than the wild-type controls (*mrck-1/+; MLC-4DD)*. We hypothesize that this is because overexpression of the constitutively active phosphomimetic MLC-4 does not perfectly recapitulate MLC-4 phosphorylation by MRCK-1 in the canal, either due to the levels, spatial organization, or timing of MLC-4 phosphorylation. We believe that the ability of MLC-4DD to significantly increase the canal lengths of *mrck-1* mutants, combined with the phenotypic similarities of the MRCK-1^{canal-} and MLC-4^{canal-} mutants provide strong evidence for phosphorylation of *mlc-4* downstream of *mrck-1.*

12) Consider adding a supplemental figure to show where the ZF tags were added to mrck-1 and mlc-4 and to clarify that all isoforms (particularly of mrck-1) were tagged using this method.

Figure S4 added to show where ZF1-tags were added to *mrck-1* and *mlc-4*

13) I think the data shown in figure 7 would fit better if incorporated into figure 6, in a parallel format to the data shown in figure 4. A summary model could be included as a new figure 7.

We agree. Figure 7 has been added to Figure 6

14) Consider clarifying that the initial characterization of EC length was made in L4/adult worms in figure 1 in the text description on page 6 and/or clarifying the first sentence under the "mrck-1 is required for canal outgrowth" section on page 7 to help readers more easily follow that figure 2 is reporting on different worm stages than are reported on in figure 1.

We added the following clarifications to the manuscript "While excretory canals of adult wild-type worms extend nearly to the end of the worm (median 0.94 relative length), we found that the *mrck-1* null, *mrck-1[K112M],* and *mrck-1ΔKin* mutants had severely truncated canals that were less than half the worm's body length (median 0.44, 0.26, and 0.19 respectively)" (p.6), Figure 1 caption "Loss of *mrck-1* or mutation of its kinase domain causes severe truncations and morphological defects in the excretory canal of adult worms.", and "The previous evaluation of canal lengths in *mrck-1* mutants was performed on late L4 stage and early adult worms when the canal has fully developed." (p.7)

15) On page 9, should also cite Sallee et al., 2018 for developing tissue specific degradation using the ZF/ZIF-1 system.

Added to manuscript.

16) At the beginning of page 10, "the expanded lumen phenotype describes canals in which most of the excretory canal volume is taken up by lumen." It would be helpful to quantify the width of expanded lumens relative to controls using the existing data.

Figure S6 has been added to compare the relative lumen diameter (lumen width/canal width) of MRCK-1^{canal-} and MLC-4^{canal-} mutants versus controls. Both mutants have significantly higher relative lumen diameters compared to controls.

17) Check the manuscript for small grammatical errors. (E.g. on page 8 "it's" should be "it is", page 12 "…reporter showed enrichment growing tip…" should be "… enrichment in the growing tip").

© 2024. Published by The Company of Biologists under the terms of the Creative Commons Attribution License (https://creativecommons.org/licenses/by/4.0/).

Updated in revised manuscript.

Reviewer 3 Comments for the Author:
Fig. 2A: I suggest drawing the worms (but not the egg) to scale, to show the dramatic difference in canal length that occurs during maintenance, similar to the growth of neurons in mammals.

Figure has been updated so all worms are drawn to scale.

In Fig. 2B: As the points for the control are identical for panels B, C, and D, these 3 graphs could be condensed if necessary, to a single graph with the dots of different forms of mrck- 1 distinguished by the color and shape.
e.g. Control Null KD &[Delta]CRIB Control Null KD &[Delta]CRIB ….
L1 L2... similarly for L3, L4, adult

For simplicity we chose to keep the graphs separate, so the viewer can more easily notice the trends for each genotype, but we have moved the graph legends to condense the graphs.

Fig. 4a: What is the control strain? Is it simply a wild-type worm? Or is it a worm expressing the ZIF-1 protein in the canal but not having the ZF1 recognition motif attached to the mrck- 1 gene. The latter would be a better control to ensure that canal-expressed ZIF-1 does not affect canal growth or morphology.

We did use worms expressing the ZIF-1 protein in the canal as our controls for Figure 4. The following clarification has been added to the legend of Figure 4: “Controls are worms carrying the *t28h11.8p::yfp::sl2::ifb-1::cfp* canal marker and *t28h11.8p::zif-1* canal-specific ZIF-1 transgenes (WD982).” We added similar notes to the end of each legend clarifying the genotypes for the controls used in each figure.

Very minor suggestions:

The grammarian says: “Wild type” is correct when “type” is a noun, but when “type” is modifying another word, the correct form is hyphenated, e.g. “wild-type worm” or “wild-type protein.” (Similarly for “dominant negative” vs. “dominant-negative mutation” or “dominant-negative fashion.”

Thanks! This has been corrected in manuscript.

Readers may confuse “KD mutants” with “KnockDown”; consider another abbreviation such as “KinD” for “Kinase-Dead” mutants.

We have renamed the allele *mrck-1[K112M]* in the manuscript (instead of ‘kinase dead’).

p.12 line 3: change “showed enrichment growing tip” to “showed enrichment **at** the growing tip”

Corrected in revised manuscript.

p. 13, last paragraph of the Results section, 8th line: change “…similar to the development expanded and convoluted…” to “…similar to the **of** expanded and convoluted…”

Corrected in revised manuscript.

For clarity, put a page break after each figure legend, so that., e.g. Figure Legend 6 is not juxtaposed next to Figure 7, and Supp. Figure Legend S3 is not juxtaposed to Supp. Figure S4.

Updated in manuscript.

Supp. Fig. S4 should have asterisks to denote the cell body in the L1 control figures.

© 2024. Published by The Company of Biologists under the terms of the Creative Commons Attribution License (https://creativecommons.org/licenses/by/4.0/).

The cell body of the canal of the control is out of frame so we added the following clarification in the figure caption: “White asterisks denote the cell body of the excretory canal (out of frame for the control).”

Second decision letter

MS ID#: DEVELOP/2024/202772

MS TITLE: MRCK-1 activates non-muscle myosin for outgrowth of a unicellular tube in *Caenorhabditis elegans*

AUTHORS: Evelyn M Popiel, Rhea Ahluwalia, Stefan Scheutz, Bin Yu, and William Brent Derry

I have now received the referee's report on the above manuscript, and have reached a decision. The referee's comments are appended below, or you can access them online: please go to BenchPress and click on the 'Manuscripts with Decisions' queue in the Author Area.

The overall evaluation is positive and we would like to publish a revised manuscript in Development. However, the reviewer provides recommendations to further improve the clarity of the manuscript. While I do not expect to send this back to the reviewers, I will look at it myself. Thus, I ask that you please attend to all of the reviewers' comments in your revised manuscript and detail them in your point-by-point response. If you do not agree with any of their criticisms or suggestions explain clearly why this is so.

Reviewer 3

Advance summary and potential significance to field

The revised manuscript by Popiel et al. provides stronger evidence and important controls that shore up the conclusion that in C. elegans, MRCK-1 (homologue to Myotonic dystrophy-Related, CDC42-binding Kinase) regulates MLC-4 (homologue to Myosin Light Chain) as necessary steps for directed outgrowth of the excretory canals, a model for formation and regulation of morphology of single-cell hollow tubes. They also show that the CDC-42-interacting “CRIB” domain of MRCK-1 is not required for this growth; this is surprising since others have shown this CRIB domain to be necessary for generating force that increases cortical tension, and other mutants affecting CDC-42 activity alter canal outgrowth and diameter. Finally, the authors show that, in many cases, the normal concomitant extension of the luminal surface is no longer tied to outgrowth of the canal cell basolateral surface. This result is consistent with the luminal and basolateral surface becoming disconnected from each other, as seen in electron micrographs of electron-dense material between the lumen and canal tip in wild-type animals (work of David Hall, at Wormatlas.org) and in mutants affecting the exc-6 gene (Shaye & Greenwald, 2016). In the present work, Fig. 5 clearly shows that even when inactive, MLC-4 at the tip of the cytoplasm remains concentrated at the growing cytoplasmic tip of the canals, separated from the endosomal marker RAB-8 located primarily near the lumen.

Comments for the author

I believe that the conclusions of this work should be of interest to a wide range of researchers who study actin-guided outgrowth in a wide range of organisms, including vertebrates, as the results imply that the CRIB domain of MRCK-1 is not the sole manner by which MRCK-1 activates MLC-4. There are a very few minor typos/corrections, however:

Fig. 1 (I-L) The yellow arrowheads indicate the end of the lumenized portion of the canal, while the white arrowheads indicate the end of the canal cytoplasm (opposite of what is stated in the figure legend); and some of the arrowheads are missing and should be included in these panels.
Line 203, “Figure 3B-E” not “Figure 2 B-E”

© 2024. Published by The Company of Biologists under the terms of the Creative Commons Attribution License (https://creativecommons.org/licenses/by/4.0/).

Line 208, “Figure 3F-h” not “Figure 2 F-H”

Second revision

Author response to reviewers' comments

We have made the requested text edits and changes to figure 1.

Third decision letter

MS ID#: DEVELOP/2024/202772

MS TITLE: MRCK-1 activates non-muscle myosin for outgrowth of a unicellular tube in *Caenorhabditis elegans*

AUTHORS: Evelyn M Popiel, Rhea Ahluwalia, Stefan Scheutz, Bin Yu, and William Brent Derry

I am happy to tell you that your manuscript has been accepted for publication in Development, pending our standard publication integrity checks.

© 2024. Published by The Company of Biologists under the terms of the Creative Commons Attribution License (https://creativecommons.org/licenses/by/4.0/).
